# Nanocomposite alginate hydrogel loaded with propranolol hydrochloride kolliphor^®^ based cerosomes as a repurposed platform for *Methicillin-Resistant Staphylococcus aureus*-*(MRSA)*-induced skin infection; in-vitro, ex-vivo, in-silico, and in-vivo evaluation

**DOI:** 10.1007/s13346-024-01611-z

**Published:** 2024-05-18

**Authors:** Moaz A. Eltabeeb, Raghda Rabe Hamed, Mohamed A. El-Nabarawi, Mahmoud H. Teaima, Mohammed I. A. Hamed, Khaled M. Darwish, Mariam Hassan, Menna M. Abdellatif

**Affiliations:** 1 Department of Industrial Pharmacy, College of Pharmaceutical Sciences and Drug Manufacturing, Misr University for Science and Technology, Giza, Egypt; 2 Department of Pharmaceutics and Industrial Pharmacy, Faculty of Pharmacy, Cairo University, Cairo, Egypt; 3 Organic and Medicinal Chemistry Department, Faculty of Pharmacy, Fayoum University, Faiyum, Egypt; 4 Department of Medicinal Chemistry, Faculty of Pharmacy, Suez Canal University, Ismailia, 41522 Egypt; 5 Department of Microbiology and Immunology, Faculty of Pharmacy, Galala University, New Galala City, Suez, 43511 Egypt; 6 Department of Microbiology and Immunology, Faculty of Pharmacy, Cairo University, Cairo, 11562 Egypt

**Keywords:** Antibacterial, Alginate nanocomposite, Biofilm, Confocal laser microscopy, In-silico study, In-vivo study, Propranolol hydrochloride, Topical drug delivery, *MRSA* skin infection

## Abstract

Nanocomposite alginate hydrogel containing Propranolol hydrochloride (PNL) cerosomes (CERs) was prepared as a repurposed remedy for topical skin *Methicillin-Resistant Staphylococcus aureus (MRSA)* infection. CERs were formed via an ethanol injection technique using different ceramides, Kolliphores^®^ as a surfactant, and Didodecyldimethylammonium bromide (DDAB) as a positive charge inducer. CERs were optimized utilizing 1^3^. 2^2^ mixed-factorial design employing Design-Expert^®^ software, the assessed responses were entrapment efficiency (EE%), particle size (PS), and zeta potential (ZP). The optimum CER, composed of 5 mg DDAB, ceramide VI, and Kolliphor^®^ RH40 showed tubular vesicles with EE% of 92.91 ± 0.98%, PS of 388.75 ± 18.99 nm, PDI of 0.363 ± 0.01, and ZP of 30.36 ± 0.69 mV. Also, it remained stable for 90 days and manifested great mucoadhesive aspects. The optimum CER was incorporated into calcium alginate to prepare nanocomposite hydrogel. The ex-vivo evaluation illustrated that PNL was permeated in a more prolonged pattern from PNL-loaded CERs nanocomposite related to PNL-composite, optimum CER, and PNL solution. Confocal laser scanning microscopy revealed a perfect accumulation of fluorescein-labeled CERs in the skin. The in-silico investigation illustrated that the PNL was stable when mixed with other ingredients in the CERs and confirmed that PNL is a promising candidate for curing *MRSA*. Moreover, the PNL-loaded CERs nanocomposite revealed superiority over the PNL solution in inhibiting biofilm formation and eradication. The PNL-loaded CERs nanocomposite showed superiority over the PNL-composite for treating *MRSA* infection in the in-vivo mice model. Histopathological studies revealed the safety of the tested formulations. In conclusion, PNL-loaded CERs nanocomposite provided a promising, safe cure for *MRSA* bacterial skin infection.

**Graphical Abstract:**

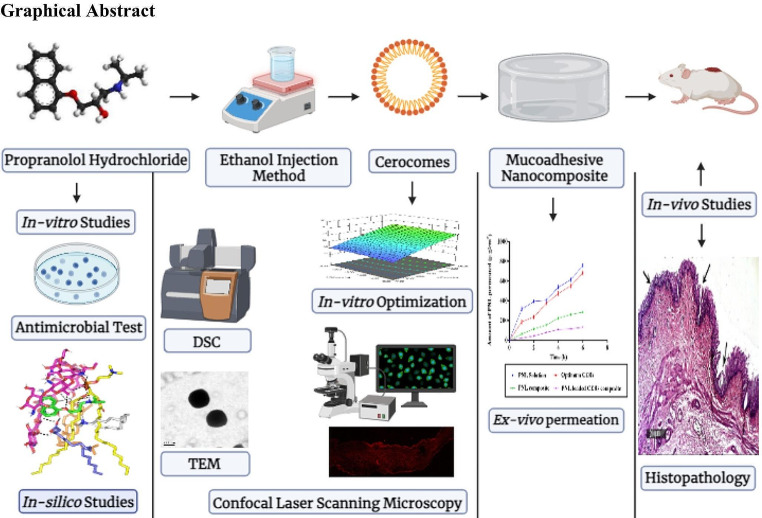

**Supplementary Information:**

The online version contains supplementary material available at 10.1007/s13346-024-01611-z.

## Introduction

Healthy skin acts as the body’s physical protective barrier from external factors such as bacteria, chemicals, and temperature. The main barrier function of the skin is related to the presence of the stratum corneum (SC) which is made up of corneocytes surrounded by lipid regions. These lipids are mainly composed of ceramides. When the skin becomes infected this might reduce the barrier function of SC resulting in increased permeability [1]. Eradication of the relevant pathogens demands efficient antibiotic treatment against the most likely microorganism [2].

Ceramides are known as the most lipophilic and the simplest kind of sphingolipids that give the SC layer its barrier function [3]. Various studies have shown that several skin conditions like psoriasis and topical infections are correlated to changes in ceramide content [4]. A few investigations reported that preparations comprising optimal ceramide concentration may help in the re-establishment of the SC layer and hence, enhance the protection property of the skin [5]. Earlier, it was demonstrated that sphingoid bases (a basic component of ceramides) may play a protective role in preventing skin colonization and infection with gram-positive organisms such as *Staphylococcus aureus* [6].

It is worth noting that the occurrence of multiple resistant *Staphylococcus aureus* skin infections has grown. These multi-resistant strains show frequent resistance to erythromycin and tetracycline, with promptly developing resistance to quinolones [7]. Bacterial resistance to antibiotics has lately grown and induced researchers to investigate diverse antimicrobial therapeutic procedures that are used to generate effective antibacterials such as drug repurposing.

Drug repurposing is a way to discover new uses for licensed and FDA-approved medicines. There are various advantages of using this method compared to developing new medications. Firstly, it has already been established that the repurposed medication is safe. Second, the time required for medication development might be decreased as the assessment of the pre-clinical studies will already have been completed. Finally, less finance is needed [8]. Several research papers are focused on developing repurposed antibacterials. For example, antipsychotics (such as Promazine) could interact with bacterial cell membranes and inhibit *Methicillin-Resistant Staphylococcus aureus (MRSA), Klebsiella*, and *pneumoniae* growth [9]. Further, antihyperlipidemic (simvastatin, pravastatin, rosuvastatin, and atorvastatin) could inhibit both *Staphylococcus aureus*, and *Streptococcus pneumoniae* (by reduction in cell viability, apoptosis promotion, bacterial membrane disruption, and protein synthesis inhibition) [10]. Moreover, a previous investigation confirmed that βeta-blockers such as propranolol hydrochloride (PNL) are effective as anti-bacterial against *Staphylococcus aureus* [11].

The efficiency of antimicrobials to eradicate bacteria from the skin’s surface depends on their efficiency on both the surface and the underlying SC. Almost all antimicrobials efficiently eliminate bacteria from the skin’s surface but not from the SC due to its tight junction [12]. Furthermore, the primary difficulty with topical medications is their ability to penetrate the skin’s deeper layers through SC to achieve efficient treatment. On the other hand, shifting from conventional therapy to high-tech-based nanomaterial therapy might be one of the most effective strategies for managing bacterial infections. Nanocarriers (NC) operate by inhibiting bacterial defense against drug resistance and the formation of biofilms or other crucial processes related to a bacterium’s potential for antipathy [13]. In addition, NC could penetrate the bacterial cell wall and membrane and interfere with essential molecular processes [14].

Numerous NCs have been developed recently, including polymeric lipid hybrid nanoparticles, nanoemulsions, and nanostructured lipid carriers, via diverse approaches and for various purposes [15]. Cerosomes (CERs) are tubular vesicles comprising ceramide prepared utilizing various surface-active agents and phospholipids (PC). When used topically they exhibit excellent permeability, increased bioavailability, and skin tolerability. The usage of surfactants in the preparation of CERs might produce stable non-aggregated lipidic vesicles [16]. It is worth noting that CERs have been reported by Abdelgawad et al. as successful carriers for the topical delivery of tazarotene to treat psoriasis [17]. Also, another investigation carried out by Albash et al. supported the efficacy of CERs topical delivery of fenticonazole nitrate for the management of *Candida albicans* infection [18].

Hydrogels are cross-linked hydrophilic polymers arranged in three-dimensional (3D) networks that can hold a lot of water while maintaining the polymer integrity and structure. Hydrogels were formulated utilizing biodegradable natural or synthetic polymers [19]. Alginate is utilized as a polymer for drug delivery applications because of its distinct features including non-toxicity, biocompatibility, non-immunogenicity, biodegradability, mucoadhesion, easy accessibility, and reasonable cost. Diverse drug delivery systems can be produced utilizing alginates, such as hydrogels, microparticles, and nanoparticles. Several methods can be employed to develop the carriers, depending on the intended usage and formulation properties. Because of its ease of use and non-toxic nature, ionotropic gelation is the most widely utilized technique for producing alginate-based particles, and hydrogels, among other systems [20].

Combining the advantages of hydrogel preparation technology and nanotechnology can help treat skin topical infections by adding new functionalities to the hydrogel structure. These functionalities improve drug therapeutic outcomes by controlling drug release and improving permeation [21].

As far as we know, no research has examined the potential of CERs-loaded alginate hydrogel to augment the deposition of Propranolol hydrochloride (PNL) as a repurposed drug in treating *MRSA*. Therefore, our study aimed to detect CERs’ potential in enhancing the topical retention of PNL and assess its safety. To attain this, various factors affecting CERs’ aspects were analyzed employing 1^3^. 2^2^ mixed factorial designs to select the optimum CER. DDAB amount (mg) (X_1_), ceramide type (X_2_), and Kolliphor^®^ type (X_3_) were examined as factors, whereas entrapment efficiency percentage (EE%; Y_1_), particle size (PS; Y_2_), and zeta potential (ZP; Y_3_), were examined as responses. The shape, effect of storage, and mucoadhesive aspects of the optimum CER were evaluated. Ex-vivo permeation investigations were carried out for the PNL solution, the optimum CER, PNL-composite, and PNL-loaded CERs nanocomposite to assess their permeability. The accumulation of the fluoro-labeled optimum CER within skin layers was traced employing a confocal microscope. In addition, in-vitro antibacterial and biofilm eradication were carried out. Moreover, the in-silico investigation was performed for the constituents of the optimum CER to evaluate how they were stable throughout binding. Finally, in-vivo investigations were carried out to estimate the antibacterial and safety capability of the prepared formulations.

## Materials

Propranolol hydrochloride (PNL) was purchased by El-Kahira Pharmaceutical Co. (Cairo, Egypt). Phospholipid from egg yolk (PC), fluorescein diacetate (FDA), Didodecyldimethylammonium Bromide (DDAB), and alginic acid sodium salt from brown algae (1000 to 1500 cP for 1% w/v in water at 25 ℃ viscosity) were purchased from Sigma Aldrich, USA. Kolliphors^®^ RH 40 and EL were gained from Acros Organics, Belgium. Ceramides III and VI were supplied by Evonic Co. (Germany). Ethanol, and chloroform were purchased from El-Nasr Pharmaceutical Co., Cairo, Egypt.

## Methods

### Preparation of PNL-loaded CERs

PNL-CERs were fabricated utilizing a modified ethanol injection approach [22]. In a mix of 1:1 ethanol/ chloroform (2mL), PC (100 mg), ceramides (III or VI), the surfactants (Kolliphors^®^ RH40 or EL), and DDAB in various amounts were all dissolved. The organic mix was added to a 10 mL hot (60 ℃) aqueous phase that had previously included PNL (60 mg) pre-solved. After 30 min of stirring the formed mix on a magnetic stirrer (Model MSH-20D, Germany) at 800 rpm, the produced mix was kept in the refrigerator.

### Characterization of PNL-loaded CERs

#### Determination of entrapment efficiency percentage (EE%)

1mL of the fabricated CERs was centrifuged at 4 °C for 1 h at 20,000 rpm utilizing a cooling centrifuge (Sigma 3 K 30, Germany). The supernatant (containing unentrapped PNL) was then diluted and PNL concentration was determined at λ_max_ 289 nm [23] utilizing a UV-VIS spectrophotometer (Shimadzu UV1650 Spectrophotometer, Japan). EE% was calculated utilizing the subsequent equation [24]:

1 $$\text{EE}\%= \left(\frac{\text{T}\text{o}\text{t}\text{a}\text{l}\, \text{a}\text{m}\text{o}\text{u}\text{n}\text{t}\, \text{o}\text{f}\, \text{P}\text{N}\text{L}-\text{U}\text{n}\text{e}\text{n}\text{t}\text{r}\text{a}\text{p}\text{p}\text{e}\text{d}\, \text{P}\text{N}\text{L}}{\text{T}\text{o}\text{t}\text{a}\text{l}\, \text{P}\text{N}\text{L}\, \text{c}\text{o}\text{n}\text{c}\text{e}\text{n}\text{t}\text{r}\text{a}\text{t}\text{i}\text{o}\text{n}}\right)\times\text{100}$$

#### Determination of particle size (PS), polydispersity index (PDI), and Zeta potential (ZP)

PS, PDI, and ZP were measured for the formulated CERs employing Zetasizer 2000 (Malvern Instrument Ltd., UK). Following proper dilution (10-fold with de-ionized water), the measurement was carried out. Every specimen was evaluated in triplicate and the mean value was noted.

#### Determination of the amount of drug released after 6 h Q6h (%)

In-vitro release investigations were conducted utilizing the United States Pharmacopeia (USP) dissolution apparatus (Pharma Test, Germany) with a diffusion area of 3.14 cm^2^. The cellulose membrane was placed between the compartments of the donor and receptor with one end sealed with a cellulose membrane and the other end, instead of baskets, linked to the USP dissolution apparatus shaft. Precisely measured 1 ml of the optimum CER dispersions, and a PNL solution equivalent to 6 mg was added into the donor cells. The receptor compartment was loaded with 50 mL phosphate buffer (pH 5.5) and kept at 37 ± 1 °C [25]. At an appropriate interval, 1 mL of release media was removed, and the same quantity of fresh media was placed in the receiver cell. Specimens were withdrawn at 1, 2, 3, 4, 5, and 6 h and scanned utilizing a UV-spectrophotometer at λ_max_ 289 nm.

### Mixed factorial experimental design

The mixed factorial design was utilized to estimate the impact of different independent variables when fabricating CERs. The factors evaluated were: DDAB amount (mg) (X_1_), ceramide type (X_2_), and Kolliphor^®^ type (X_3_). EE%, PS, and ZP were selected as responses (Table 1). Every parameter was submitted to an analysis of variance (ANOVA) testing employing Design-Expert^®^ (Version 13, Stat-Ease., USA). Statistical significance was detected utilizing *p*-values < 0.05.

**Table 1:** Full factorial design for optimization of PNL-loaded CERs

Factors (independent variables)	Levels			
	Low (-1)		High (+ 1)	
X_1_: DDAB Amount (mg)	0		10	
X_2_: Ceremide Type	III		VI	
X_3_: Kolliphor^®^ Type	EL		RH	
Responses (dependent variables)	**Constraints**			
Y_1_: EE (%)	Maximize			
Y_2_: PS (nm)	Minimize			
Y_3_: ZP (mV)	Maximize			

*Abbreviations* DDAB; Didodecyldimethylammonium Bromide, EE%; Entrapment Efficiency Percentage, PS; Particle Size, ZP; Zeta Potential, PNL; Propranolol Hydrochloride and CERs; Cerosomes

### Selection of the optimum PNL-loaded CER

The choice of the optimum CER was based on the function of the desirability that permitted the studying of whole responses concurrently. The aim was to attain a solution with maximum ZP and EE% and minimum PS. The suggestion with the greatest desirability solution was opted as shown in (Table 1). The optimum CER was formulated, inspected, and compared with the predicted data to make sure that the model was performing accurately.

### Transmission electron microscopy (TEM)

TEM (Joel JEM 1230, Japan) was used to determine the morphological features of the optimum CER. The dyed formulation was added to a copper-covered carbon grid and then left for drying till TEM investigations were conducted [26].

### Differential scanning calorimetry (DSC)

The thermal evaluation of PNL, the optimum CER ingredients, the physical mixture, and the optimum CER was done by utilizing differential scanning calorimetry (DSC-60, Shimadzu Corp., Japan) standardized with indium. Almost 5 mg of specimens were put into an aluminum pan using a temperature ranging from 10 to 350 ℃ at a rate of 5 ℃/min under a nitrogen stream [27].

### Muco-adhesion test

The mucoadhesive characteristics of the optimum CER were assessed utilizing porcine mucin (1% w/v) that was blended under stirring for five minutes with an equal quantity of the optimum CER in a dropwise pattern. The mix was permitted to equate at room temperature overnight then it was diluted 10-fold with de-ionized water before measuring its charge. Utilizing a zetasizer the charge of mucin particles in the presence and absence of mucoadhesive vesicles was assessed [28].

### Impact of storage

The optimum CER stability was examined to monitor vesicles’ formation, PNL leakage, or any change. The optimum CER was stored for 3 months in a refrigerator and its stability was then tested by comparing the EE%, PS, ZP, PDI, and Q6h (%) of the stored optimum CER to the newly formulated one. Furthermore, the preparation was examined for any precipitation or aggregation. Statistical significance was conducted utilizing Student’s *t*-test employing SPSS^®^ software [25].

### Preparation of alginate hydrogel composite (PNL-composite and PNL-loaded CERs nanocomposite)

Alginate hydrogel-loaded with the optimum CER was formulated utilizing the approach described in the literature [29], with some minor adjustments. Briefly, under stirring at 1500 rpm and 70 ℃ for 15 min (Hot plate magnetic stirrer., Korea), 2% w/v of sodium alginate was added to the CERs to ensure the entire dissolving of sodium alginate and a consistent dispersion of the CERs throughout the fabricated hydrogel. A precisely determined volume was removed from the prepared alginate-gel and put into circular-base vessels. Later, the same amount of anhydrous CaCl_2_ aqueous solution (0.2 M) was inserted all at once into the vessels holding the alginate gel until the development of uniform dispersal; then the mix was kept at room temperature for 10 min to allow the cross-linking process [30]. Subsequently, the fabricated hydrogels were taken out and washed with distilled water three times with a constant volume of 20 mL to get rid of any excess non-crosslinked CaCl_2_ and CERs then kept in separate containers at room temperature. To prepare PNL-alginate hydrogel (PNL-composite) and non-medicated alginate hydrogel (blank), the same procedures were followed, but distilled water was used in place of the CERs dispersion for comparison in the in-vivo investigations.

### Ex-vivo studies

#### Tissue collection

In this examination, rat skin was utilized because of its availability, small size, and low cost. First, animals were slaughtered, and the skin was eliminated. After that, an electrical clipper was utilized to remove hair from rat skin and the subcutaneous tissues and adhering fats were removed by cotton brushing. The removed skin specimens were equilibrated by immersing them in saline before starting the examination [31].

#### Ex-vivo permeation studies

This investigation aimed to examine the ability of 1 mL of PNL solution, optimum CER, PNL-composite, and PNL-loaded CERs nanocomposite gel (containing 6 mg PNL) to penetrate the rat’s skin. The specimens were added into cylindrical tubes featuring a diffusion area of 3.14 cm^2^ with one end covered with skin and the other end linked to the USP dissolution apparatus shaft (Pharma Test, Germany). The formulations were placed in 50 mL phosphate buffer (pH 5.5) at 37℃. Specimens were removed at 1, 2, 3, 4, 5, and 6 h and examined utilizing HPLC [32]. Using ANOVA test statistical significance was tested employing SPSS^®^. Post-hoc-analysis was carried out utilizing Tukey’s honestly significant difference (HSD) test. The significance was opted at *P* < 0.05.

#### Ex-vivo confocal laser scanning microscopy studies

To determine the passing of the optimum CER via the skin, the fluoro-labeled CERs were fabricated as described in the preparation section but with the inclusion of 10 mg FDA instead of PNL. Rat’s skin was placed as the ex-vivo investigation. FDA-loaded CERs were put on the dorsal skin surface and kept for 6 h. After that, longitudinal sections of skin were sliced utilizing a microtome (Cambridge, UK), and the skin tissues were then examined under an inverted microscope (Carl Zeiss, Germany) to determine the presence of fluorescence.

### In-silico studies

#### Molecular docking of PNL against multiple MRSA biotargets

PNL was constructed and energy-minimized at MMFF94s partial charges and MMFF94s-modified forcefield as previously described using MOE software package (Quebec, Canada) [33–35]. *MRSA*’s biological targets involved within peptidoglycan synthesis; MurE (PDB: 4c12), MraY (AlphaFold: Q2FZ93), FemA (PDB: 1lrz), and PBP2a (PDB: 3zg0) were deposited and structurally prepared under 3D-protonation (pH 7.4, and 0.1 M salt solution /Volumn-Integral implicit solvent model), in addition, autocorrected for atom types, partial charges, and bond connectivity. Binding sites were defined by harboring the co-crystallized ligand, explored via the MOE Alpha Site Finder geometrical method, and finally optimized for including the essential residues that were previously stated within the context.

The docking was conducted using the induced docking protocol that allowed significant pocket residue elasticity. Ligand configurations were established On-the-Fly utilizing a bond rotation/ligand placement approach, within the determined active site, and guided utilizing triangular-matcher protocol [36]. The resultant configurations were ordered utilizing London_dG and the top 10 docked poses/ligands were optimized, and energy minimized allowing the protein’s residue sidechains to be tethered. Optimized poses were subsequently rescored utilizing Generalized Born-solvation-VI/Weighed Surface-Area_dG forcefield depending on explicit solvation electrostatics, current-loaded charges, exposure-weighted surface area, and Coulombic electrostatics via protein-ligand van der Waals scores [37, 38]. High docking scores, RMSD values below 2.0 Å cut-off, and/or significant interactions with stated essential-pocket-residues were regarded for selecting the best pose. PyMol2.0.6 (Schrödinger, USA) and MOE wizard/measurement apparatus were employed for visualization.

#### Molecular modeling simulation of the nano-formulation drug complex

Computational investigation under vacuum conditions was performed by applying the AutoDock package v1.2.0 (Scripps Research Institute, California, United States). PNL and formulation additives were 2D constructed using the isomeric SMILES deposited at PubChem database (PNL ID_4946), ceramide-VI ID_44625889, PC ID_65167, (DDAB) ID_18669, and Kolliphor^®^ RH40 unit molecule ID_482024909. Constructed molecules were transformed into 3D structures and energy was reduced under AMBER/modified partial charges and forcefield. Molecular docking protocol was done utilizing Lamarckian Genetic Algorithm-driven conformational search under AMBER Forcefield [39].

Docking protocol proceeded through Triangular/Matching combined with the London ΔG-ranking scoring system as well as a refinement for the 10 top-scored poses via energy minimization under Generalized Born-solvation_VI/Weighted-SA/ΔG forcefield. The refinement forcefield scoring is generally based on van der Waals potentials, Coulomb’s interactions, loaded partial charges, polar solvation potentials, and weighed exposure SA [40]. Provided docked binding scores as binding energies, whereas choosing the best-docked pose was a combination of high docking score consideration and significant polar/hydrophobic interactions between corresponding formulation additives. PyMol v2.0.6 (Schrödinger, United States) was utilized to visualize the predicted docking results and analyze the inter-compound binding contacts. The docked drug/nano-formulation complex was set as a reference structure for all-atom molecular dynamics simulations using MOE software under the explicit Amber10: EHT forcefields [41, 42]. The 3D water cube-shaped box was used to solvate the drug-formulation complex under periodic boundary conditions with distances of 10 Å (Table 2) [43]. The system was minimized through NVT ensemble (300 K) for 50 ps, subsequently equilibrated under NPT ensemble (300 K; 1 atm. Pressure) for 50 ps. Explicit molecular dynamics simulations were then produced through 1000 ps under the NPT ensemble. Studying the MD trajectories was performed utilizing the MOE Database Calculator for plotting potential/kinetic energy versus time as well as the compound’s average binding-free energy towards the PC nano-formulation components. Time-evolution conformational changes for the drug nano-formulation were monitored across the extracted timeframes at 200 ps intervals.

**Table 2:** Atomic composition of PNL-CERs formulation for molecular dynamics simulation

Solvation State	Atomic composition (№. of atoms)						
	PNL	PC	Ceramide-VI	DDAB	Kolliphor^®^-RH40	Aqua	Entire Model
100% TIP3P water model	50	134	115	84	200	3 × 3646	11.521

*Abbreviations* DDAB; Didodecyldimethylammonium Bromide, PNL; Propranolol Hydrochloride, PC; Phospholipid, and CERs; Cerosomes

### In-vitro antibacterial activity

This investigation aimed to determine the antibacterial potency of PNL *against MRSA USA300*. Utilizing the broth microdilution approach following the guidelines of the Clinical and Laboratory Standards Institute, the minimum inhibitory concentration (MIC) was determined [44, 45].

### Anti-biofilm activity of PNL

#### Biofilm inhibition assay

Utilizing flat-bottom 96-well plates, the biofilm inhibition assay was conducted as formerly mentioned [46]. Various sub-MIC concentrations (0.3125–0.0098 mg/mL) of PNL were examined. Untreated groups (no drug was included) were opted as controls. The experiment was repeated in triplicates. The % of biofilm inhibition was computed utilizing the subsequent equation [47]:

2 $$ Biofilm\,inhibition\%= \frac{OD \,Control-OD \,Test}{OD \,Control} \times 100$$

#### Biofilm eradication assay

This investigation aimed to evaluate the capability of PNL to detach and eradicate the already formed *MRSA USA300* biofilm. The experiment was performed as described before [46]. Various sub-MIC concentrations (0.3125–0.0098 mg/mL) of PNL were formulated in fresh media and then inserted into the plates of the biofilm. For the biofilm control groups, nothing was applied to the plates (untreated biofilm, 100% reference value). The trial was conducted in triplicates. The % of biofilm eradication was computed utilizing the subsequent equation [47]:

3 $$ Biofilm\,eradication \%= \frac{OD \,Control-OD \,Test}{OD \,Control} \times 100 $$

### In-vivo studies

#### In-vivo MRSA skin infection model

All trials and animal-procedures were accepted by the Research Ethics Committee of the Faculty of Pharmacy Cairo University (Approval# MI3394) following the “Guide for the Care and Use of Laboratory Animals” published by the Institute of Laboratory Animal Research (Washington, DC, USA). The *MRSA-related* skin infections model was performed as previously reported [45, 48, 49]. Twenty-eight BALB/C male mice aged 7 weeks old were kept in cages at ambient temperature with food and water. Before the assessment, the mice’s dorsal backs were shaved and then injected intradermally with 100 µL *MRSA USA300* suspended in sterile saline (9 × 10^8^ CFU). Mice were randomly classified into 4 groups (*n* = 7). After 48 h of infection and the development of an open wound, the first group served as the negative-control. The second group was handled with a blank formula to be used as vehicle-control. The third group was handled with the PNL-composite. The fourth group was topically handled with PNL-loaded CERs-nanocomposite. All groups were topically handled at the infection site utilizing 100 µL of the assigned medication one time a day for three days. The experiment ended 24 h after the last treatment and the animals were euthanized; the skin laceration was cleaned off, and then homogenized by 0.5 ml saline. Specimens were diluted 10 folds and then they were plated on mannitol salt agar and incubated at 37 °C to determine the aerobic viable count. Following incubation for 24 h, the plates were checked for colony-forming units (CFU) and the findings of the examined groups were analyzed and contrasted.

#### Histopathologic evaluation

Fifteen mice were classified into 5 groups, each containing 3 mice, and treated for one week: group I served as negative control, group II served as positive control, group III was handled with vehicle gel, group IV was handled with PNL-composite, and group V was handled with PNL-loaded CERs nanocomposite. After the autopsy, specimens were kept in 10% formalin and dried and sectioned at 4 μm utilizing a microtome (Cambridge, UK). The samples were subsequently deparaffinized, dyed, and histopathologically evaluated employing light microscopy [48].

## Results and discussion

### Optimization of CERs using mixed factorial design

Quality by design is a powerful statistical technique that validates the procedure’s effectiveness in terms of mathematical correlations. The desirability function is an efficient method for determining the optimum levels of the variables. The two-factor interaction (2FI) model was selected because it produced the highest prediction R^2^. Adequate precision is employed to assert that the model could be used for navigating the design space [50]. A ratio (signal-to-noise ratio) greater than 4 (for adequate precision) is favored which was observed in each of the responses (except PDI) as seen in Table 3. For design-analysis outcomes (Table 3), it was obvious that the predicted R^2^ values were in good harmony with the adjusted R^2^ in each of the responses.

**Table 3:** Output data of the full factorial analysis of CERs formulations and predicted and observed values for the optimum CER

Responses	EE (%)	PS (nm)	ZP (mV)
Adequate precision	46.19	28.49	82.87
Adjusted *R*^2^	0.988	0.974	0.997
Predicted *R*^*2*^	0.978	0.953	0.995
Significant factors	(X_1,_ X_2_ and X_3_)	(X_1,_ X_2_ and X_3_)	(X_1,_ and X_3_)

*Abbreviations* DDAB; Didodecyldimethylammonium Bromide, EE%; Entrapment Efficiency Percentage, PS; Particle Size, ZP; Zeta Potential, and CERs; Cerosomes

#### Effect of formulation variables on the EE%

**Fig. 1:**
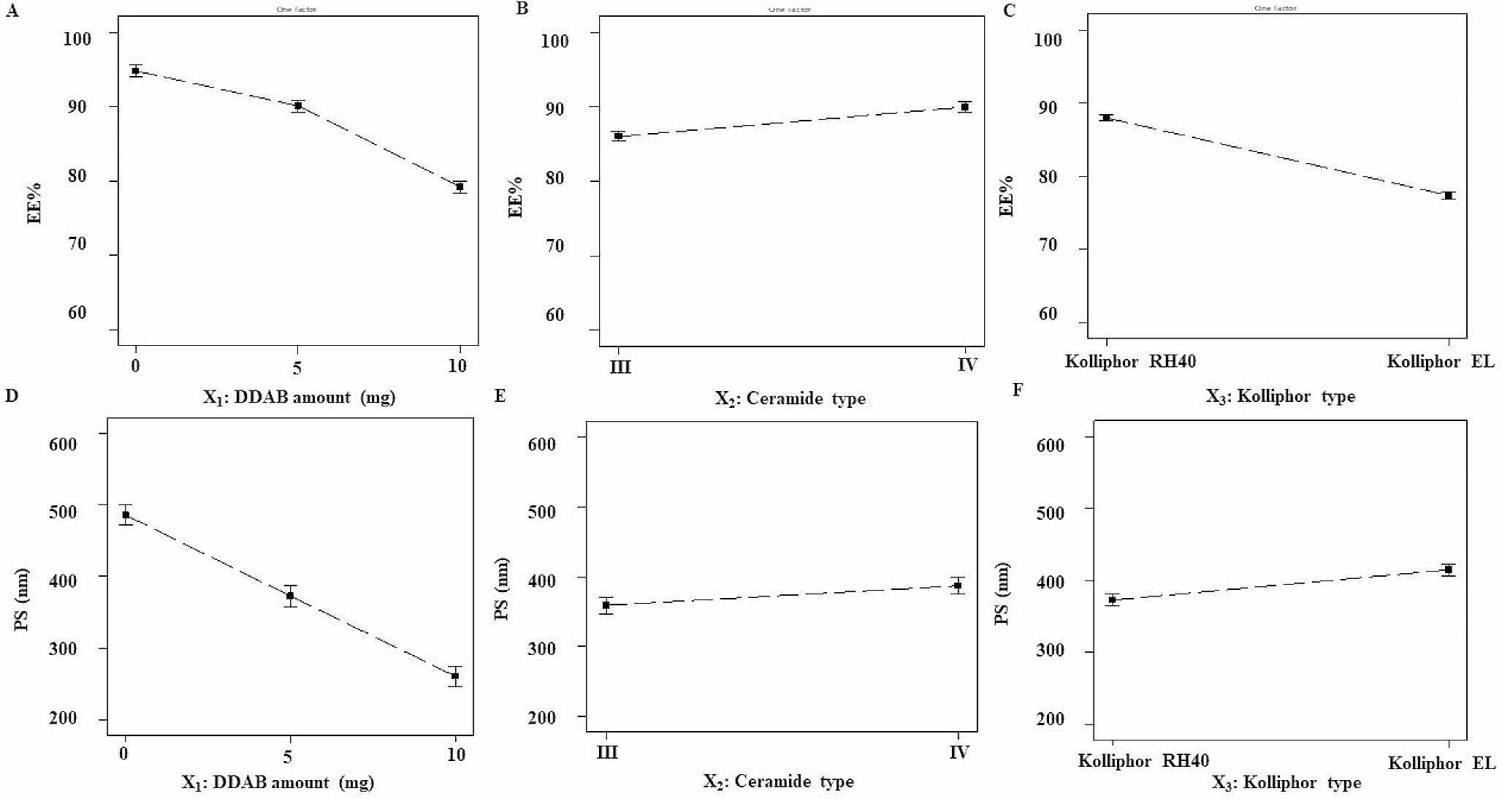
Effect of formulation variables on EE% of PNL-CERs (**A**-**C**), and PS (**D**-**F**). *Abbreviations* EE%; Entrapment Efficiency Percentage, PNL; Propranolol Hydrochloride, DDAB; Didodecyldimethylammonium Bromide, PS; Particle Size, and CERs; Cerosomes

EE% of the formulated PNL-loaded CERs ranged from 65.71 ± 0.95% to 96.06 ± 0.97% (Table 4). The relatively great EE% values from the prepared CERs might be related to the presence of ceramide that enhances the nano-dispersions’ viscosity, impeding the drug diffusion, and leading to greater EE% values [26]. Statistical analysis of the data utilizing ANOVA showed that all the investigated variables had a significant impact on the EE% (Fig. 1(A–C)). Considering the DDAB amount (mg) (X_1_) (*p* < 0.0001) it was obvious that by augmenting the DDAB amount the EE% decreased significantly. This might be related to the DDABs’ improvement of PC solubility, which encourages the leakage of PNL from CERs. Conversely, the greater EE% values at low DDAB amounts might be attributed to the formation of tight bilayers around PNL and insufficient surface-active agent (SAA) amounts to solubilize PC bilayers [28].

**Table 4:** Experimental runs, independent variables, and measured responses of the full factorial design of PNL-loaded CERs

F	DDAB Amount (mg) (X_1_)	Ceramide Type (X_2_)	Kolliphor^®^ Type (X_3_)	EE (%)	PS (nm)	PDI	ZP (mV)
F1	0	III	RH40	93.50 ± 1.44	457.05 ± 14.74	0.365 ± 0.08	4.79 ± 0.05
F2	0	VI	RH40	96.06 ± 0.97	515.22 ± 15.68	0.357 ± 0.06	8.52 ± 0.01
F3	0	III	EL	83.72 ± 0.67	576.70 ± 19.98	0.354 ± 0.04	4.30 ± 0.11
F4	0	VI	EL	88.95 ± 0.42	586.42 ± 14.46	0.365 ± 0.01	1.87 ± 0.06
F5	5	III	RH40	87.97 ± 0.43	370.15 ± 18.14	0.358 ± 0.07	22.96 ± 1.89
F6	5	VI	RH40	92.91 ± 0.98	388.75 ± 18.99	0.363 ± 0.05	30.36 ± 0.69
F7	5	III	EL	75.80 ± 0.59	383.84 ± 25.58	0.367 ± 0.05	22.04 ± 0.09
F8	5	VI	EL	80.88 ± 0.55	405.56 ± 15.73	0.356 ± 0.06	26.79 ± 0.17
F9	10	III	RH40	76.62 ± 0.75	250.34 ± 17.51	0.359 ± 0.09	32.84 ± 0.06
F10	10	VI	RH40	81.70 ± 1.80	270.83 ± 17.98	0.364 ± 0.02	37.88 ± 1.08
F11	10	III	EL	65.71 ± 0.95	253.98 ± 19.37	0.370 ± 0.01	31.12 ± 0.79
F12	10	VI	EL	69.18 ± 0.89	283.83 ± 11.69	0.358 ± 0.03	34.98 ± 1.26
Observed values for CER6				92.91	388.75		30.36
Predicted values for CER6				92.27	381.32		30.53
Bias%				0.69	1.91		0.56

*Note* Data represented as mean ± SD (*n* = 3). Abbreviations: DDAB; Didodecyldimethylammonium Bromide, EE%; Entrapment Efficiency Percentage, PS; Particle Size, ZP; Zeta Potential, PDI; Polydispersity Index, PNL; Propranolol Hydrochloride, and CERs; Cerosomes

Regarding ceramide type (X_2_) (*p* < 0.0001) it was found that the EE% increased by using ceramide VI compared to ceramide III. As ceramide VI consists of a saturated phytosphingosine structure acylated with hydroxy stearic acid long chain [51]. On the other hand, ceramide III is composed of a phytosphingosine backbone acylated with oleic acid in addition, it has one unsaturated bond in its fatty acid chain [5] the previously observed unsaturated double-bond in the carbon chain may affect it to twist, making CERs leakier since the packing of the CERs may not be tight [27].

For Kolliphor^®^ type (X_3_) (*p* < 0.0001), it was found that Kolliphor^®^ RH 40 produced higher EE% values compared to Kolliphor^®^ EL. This might be ascribed to the presence of unsaturation sites in the alkyl chains of Kolliphor^®^ EL that may enhance the permeability of the vesicle membrane, consequently diminishing EE% [52]. The existence of the unsaturated double-bond in the chain of carbon as previously stated might cause twisting that promotes leaking from CERs as the packing of the CERs might not be firm. These findings were consistent with Abdelbary et al. who found that bilosomes containing Kolliphor^®^ RH 40 entrapped more terconazole than those containing Kolliphor EL [53]. On the other hand, Kolliphor^®^ RH 40 has a higher molecular weight than Kolliphor^®^ EL, previous literature stated that higher molecular weight SAA might aid in steric stabilization and keep the nanocarriers from aggregating or fusing. This may also result in a greater degree of trapping effectiveness [54].

#### Effect of formulation variables on PS

The size of the nanocarriers plays a prime role in improving the rate of dissolution as well as in their adhesion and interaction with biological cells [55]. PS of the fabricated PNL-loaded-CERs ranged from 250.34 ± 17.51 to 586.42 ± 14.46 nm (Table 4). Statistical analysis of the data utilizing ANOVA showed that all the investigated variables had a significant impact on the PS (Fig. 1 (D–F)). Considering the DDAB amount (mg) (X_1_) (*p* < 0.0001) it was apparent that by increasing the DDAB amount the PS decreased significantly. The prior findings were consistent with Abdellatif et al. [56] who discovered that the increase in cationic SAA produces steric repulsion, which prevents or decreases vesicle aggregation. Further, the increase in DDAB might diminish the interfacial tension between aqueous lipids, resulting in the formation of smaller vesicles, or due to surfactants’ lipid solubilization, resulting in a smaller PS.

Regarding ceramide type (X_2_) (*p* = 0.0076), the PS was greater in CERs contained VI compared to CERs contained III the previous results agreed with EE% results as higher PS CERs were found to be with CERs with higher EE% [57]. On the other hand, ceramide III and ceramide VI molecular weights are 582 and 600, respectively. Although there was a slight difference in the molecular weight of ceramides, it resulted in the development of larger CERs in the case of CERs produced by ceramide VI. As previously reported, the rise in the molecular weight might augment the viscosity of the nano-dispersion developing aggregations and augmenting the PS [58].

For Kolliphor^®^ type (X_3_) (*p* < 0.0001), it was found that Kolliphor^®^ RH 40 produced smaller PS values compared to Kolliphor^®^ EL. Both SAAs are made up of hydrophilic and hydrophobic counterparts. The hydrophilic counterparts of both SAAs are accountable for preventing vesicle aggregation. They are made of polyethylene oxide (PEO) units with 40 PEO units for Kolliphor^®^ RH 40 and only 35 PEO units for Kolliphor^®^ EL [58]. Consequently, the employment of the former Kolliphor^®^ which has a more stabilizing ability led to the formation of smaller PS, the previous findings agreed with previous literature [59].

#### Evaluation of PDI

It is commonly known that sample heterogeneity is indicated by PDI values close to 1, whereas values close to 0 indicate size homogeneity [60]. The observed values (Table 4) ranged from 0.356 ± 0.01 to 0.370 ± 0.01. These confirmed the evaluated samples’ relative homogeneity as the ethanol injection method usually produces a homogenous population [61].

#### Effect of formulation variables on ZP

**Fig. 2:**
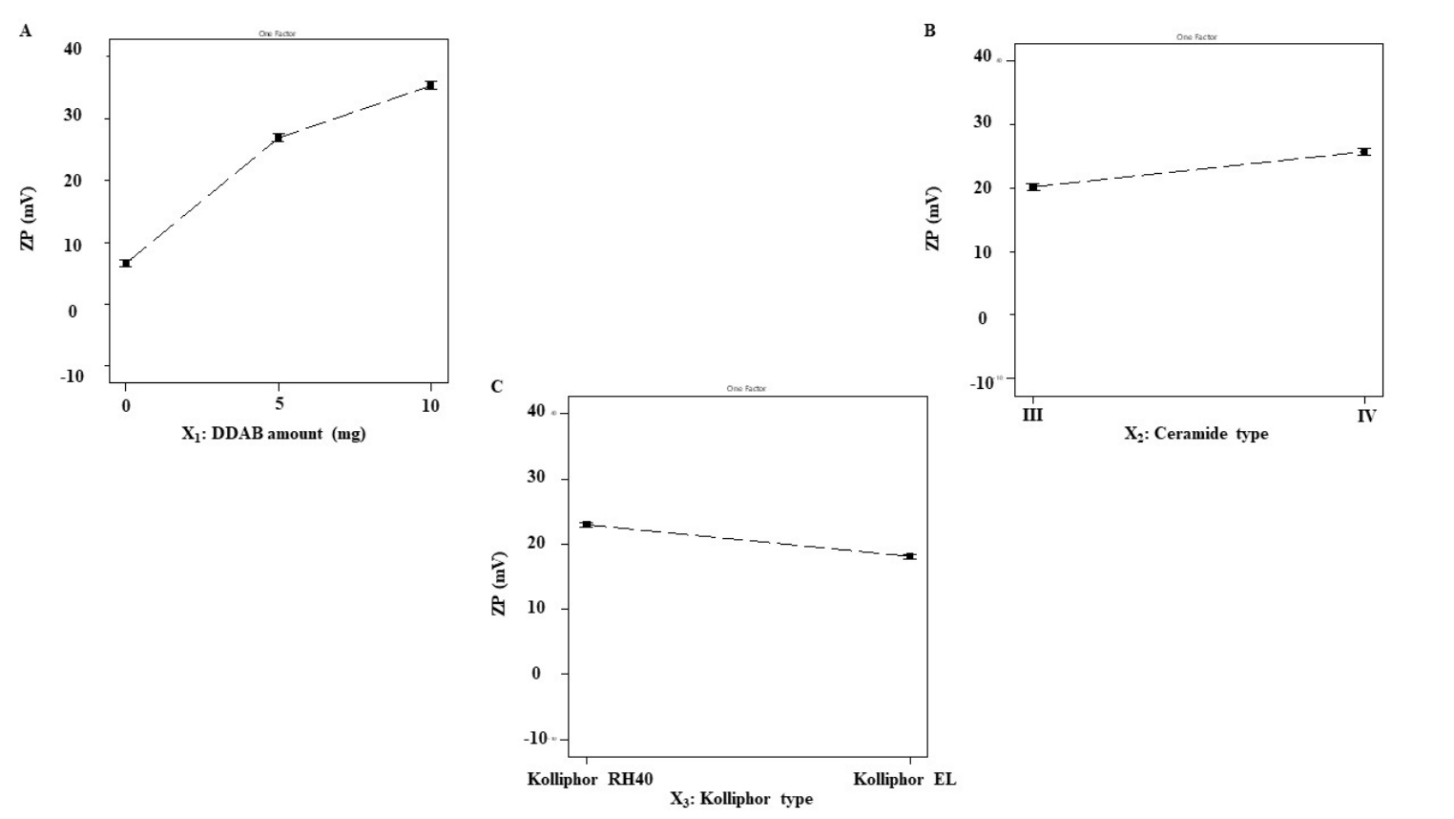
Effect of formulation variables on ZP of PNL-CERs. *Abbreviations* ZP; Zeta Potential, DDAB; Didodecyldimethylammonium Bromide, PNL; Propranolol Hydrochloride, and CERs; Cerosomes

The ZP values ranged from 1.87 ± 0.06 to 37.88 ± 1.08 mV (Table 4). Because of the negatively charged protein residues that are present on the surface of human skin, the skin is negatively charged [62]. Hence, the positive charge in the skin nano-formulations is preferred to augment electrostatic interaction between cationic formulations as presented in CERs and the skin surface, leading to greater retention of the drug in skin layers. The resulting data (Table 4) and (Fig. 2 (A-C)) reveal the impact of the investigated variables on the ZP of the CERs.

Considering the DDAB amount (mg) (X_1_) (*p* < 0.0001) it was evident that by increasing the DDAB amount the ZP increased significantly. The existence of the cationic surfactant (DDAB) affected the fabricated CERs-ZP values as it reduced the surface charge’s negativity. Further, the increase in ZP could be ascribed to the existence of DDAB molecules in high amounts at the interface of CERs, resulting in greater positive ZP values [63].

For Kolliphor^®^ type (X_3_) (*p* < 0.0001), it was found that Kolliphor^®^ RH 40 produced higher ZP values compared to Kolliphor^®^ EL. Kolliphor^®^ RH40 is more hydrophilic (HLB ∼ 15) than Kolliphor^®^ EL (HLB ∼ 13), which helps the dispersion of the lipids in the aqueous phase [64]. A previous study stated as the HLB of the SAA increases it usually produces a more stable nanosystem with higher ZP values [64].

#### Determination of the optimum CER

The optimum CER from the experimental design was CER6 which met the previously established criteria (maximum ZP and EE% and minimum PS). It was selected utilizing Design Expert software^®^ version 13. The observed and predicted values of CER6 revealed a high correlation (Table 3). Also, the high predictive capacity of the model was demonstrated by the bias percent values, which were lower than 10% for all the evaluated responses [65]. Subsequently, CER6 was subjected to extra studies.

#### In-vitro drug release

**Fig. 3:**
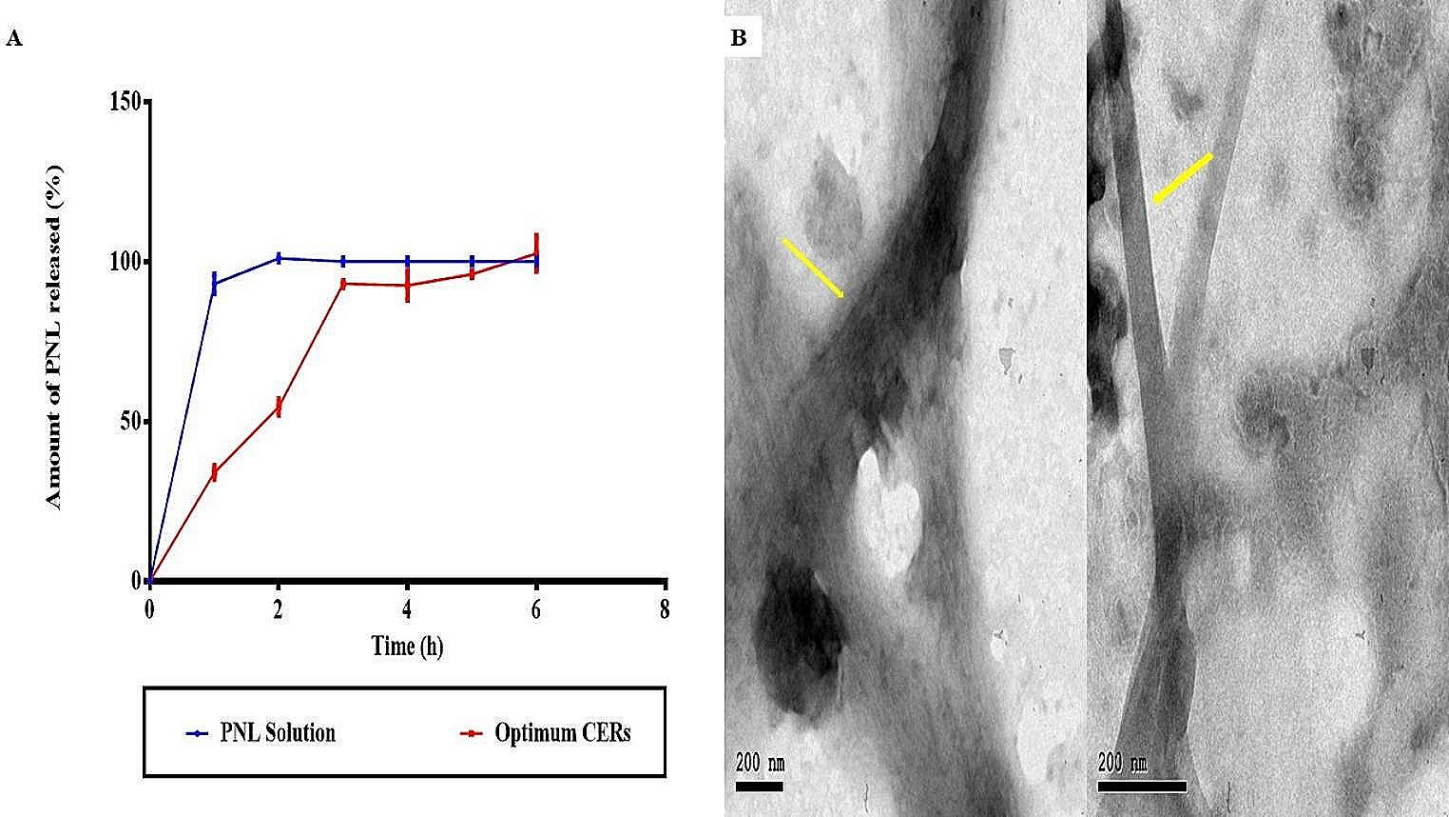
Amount of PNL released after 6 h Q6h (%) for PNL solution and optimum CER (**A**), and Transmission electron micrographs of the optimum CER (**B**). *Abbreviations* PNL; Propranolol Hydrochloride, and CERs; Cerosomes

NCs have lately proven probable utility in the treatment of bacterial infections through their topical application that enables controlled and long-lasting release. Due to their small size and the possibility of modifying their surface charge, they can biologically interact with the body systems and hence, improve their in vivo therapeutic efficacy [65, 66]. From Fig. 3A, it was found that Q6h (%) for the optimum CER was 86.60 ± 5.5% and the release pattern of PNL from the optimum CER was at first rapid and then significantly (*p* = 0.0001) changed to sustained compared to the PNL solution where PNL was entirely released from the PNL solution after 2 h. The prior findings might be ascribed to the following reasons, firstly: PNL being an amphoteric drug, it has affinity toward the aqueous phase. Secondly: the presence of both PC and ceramide could offer a depot property that contributes to the continual release of PNL in a sustained manner from CERs for fighting against bacterial infection [67].

### Transmission electron microscopy (TEM)

TEM investigation (Fig. 3B) revealed that ceramide addition to the composition of the CERs led to a dramatic change in the morphology of their membrane from spherical to tubular [17]. In the TEM image of CERs, the prevalent morphological form was elongated ceramide tubules, with vesicles appearing less frequently. This was also confirmed by Xu et al. [51] who found that adding ceramide to PC resulted in the elongation of the fabricated vesicles which was ascribed to the partitioning of ceramide VI into the PC bilayer associated with the interface rigidification. Also, the high packing parameter of ceramide (1.2) in comparison to that of the PC (0.7) made the PC bilayer curvature flattened upon hydration with an aqueous solution [51]. The irregular existence of tubules along with the spherical vesicles as shown in the optimum CER resulted in a distinctive bulbous feature of the tubules. It was confirmed by the authors who stated that ceramide VI tends to distribute in a non-uniform design in the bilayer, resulting in ceramide-rich areas with a flat shape and ceramide-poor areas with a spherical shape.

### Differential scanning calorimetry (DSC)

**Fig. 4:**
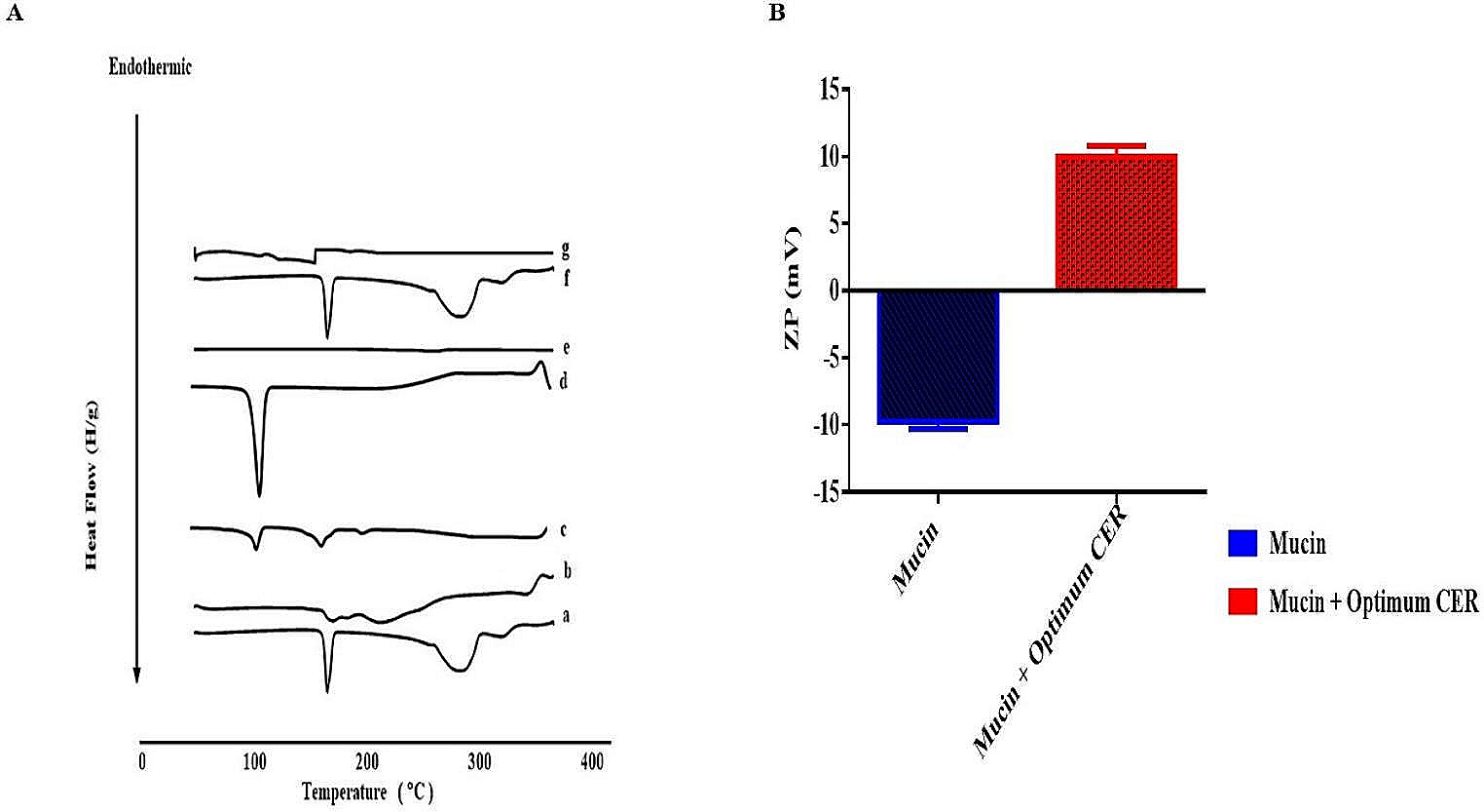
Differential scanning calorimetry study (**a**) PNL, (**b**) PC, (**c**) ceramide VI, (**d**) DDAB, (**e**) Kolliphor RH40, (**f**) physical mixture, and (**g**) optimum CER (**A**), and Mucoadhesion test for the optimum CER (**B**). *Abbreviations* CER; Cerosome, DDAB; Didodecyldimethylammonium Bromide, and PNL; Propranolol Hydrochloride

From Fig. 4A, PNL showed an endothermic peak representing its melting point at 166.91℃ [25]. In addition, PC showed two endothermal peaks. The first peak (159.9 °C) was described as mild, showing hot movements of polarity parts of PC. The second peak manifested at 234.6 °C, which might be attributed to the transition from gel to liquid crystal state, the melting of the carbon-hydrogen chain in PC, and changes in isomers or the crystal [25]. Ceramide VI showed an exothermic peak at 100.3℃ [68]. Moreover, the thermogram of DDAB showed a sharp peak at 110℃ [69]. The thermogram of Kolliphor^®^ RH40 showed no exothermal or endothermal peaks [70]. The DSC of the physical mixture of PNL with CERs constituents disclosed that the PNL peak was existent supporting its presence in the crystalline state. The thermogram of the optimum CER did not reveal the melting peak for PNL. This manifested that PNL was in an amorphous state, and it was completely entrapped into the CER [71]. In addition, the disappearance of a distinctive peak of PNL might also suggest a major interaction between the drug and the constituents of the CER structure which might be the reason for the high EE% of PNL. These interactions could account for the good vesicle shape, structure, and excellent stability.

### Mucoadhesion test

To investigate the mucoadhesive properties of the investigated optimum CER, the mucin solution was mixed with the examined formulation. Results revealed a significant change in the ZP value of pure mucin solution from − 7.99 ± 1.20 to 10.11 ± 0.09 mV after being mixed with the tested formulation (Fig. 4B). This change might have resulted from the interaction of the negatively charged sialic-acid found in mucin with the positively-charged-amino groups of the utilized cationic SAA (DDAB). It is worth mentioning that DDAB is a quaternary ammonium SAA that possesses a central ammonium group with a permanent positive charge attached to double alkyl chains. In addition, DDAB has an amphiphilic character due to the presence of both a polar head and hydrophobic tail, respectively present in its structure. This ionic interaction indicated the mucoadhesive properties of CERs. These mucoadhesive features can prolong the duration that the applied formulation remains on the skin, hence augmenting the local concentration of the drug at the application site and enhancing the drug’s bioavailability.

### Impact of storage

**Fig. 5:**
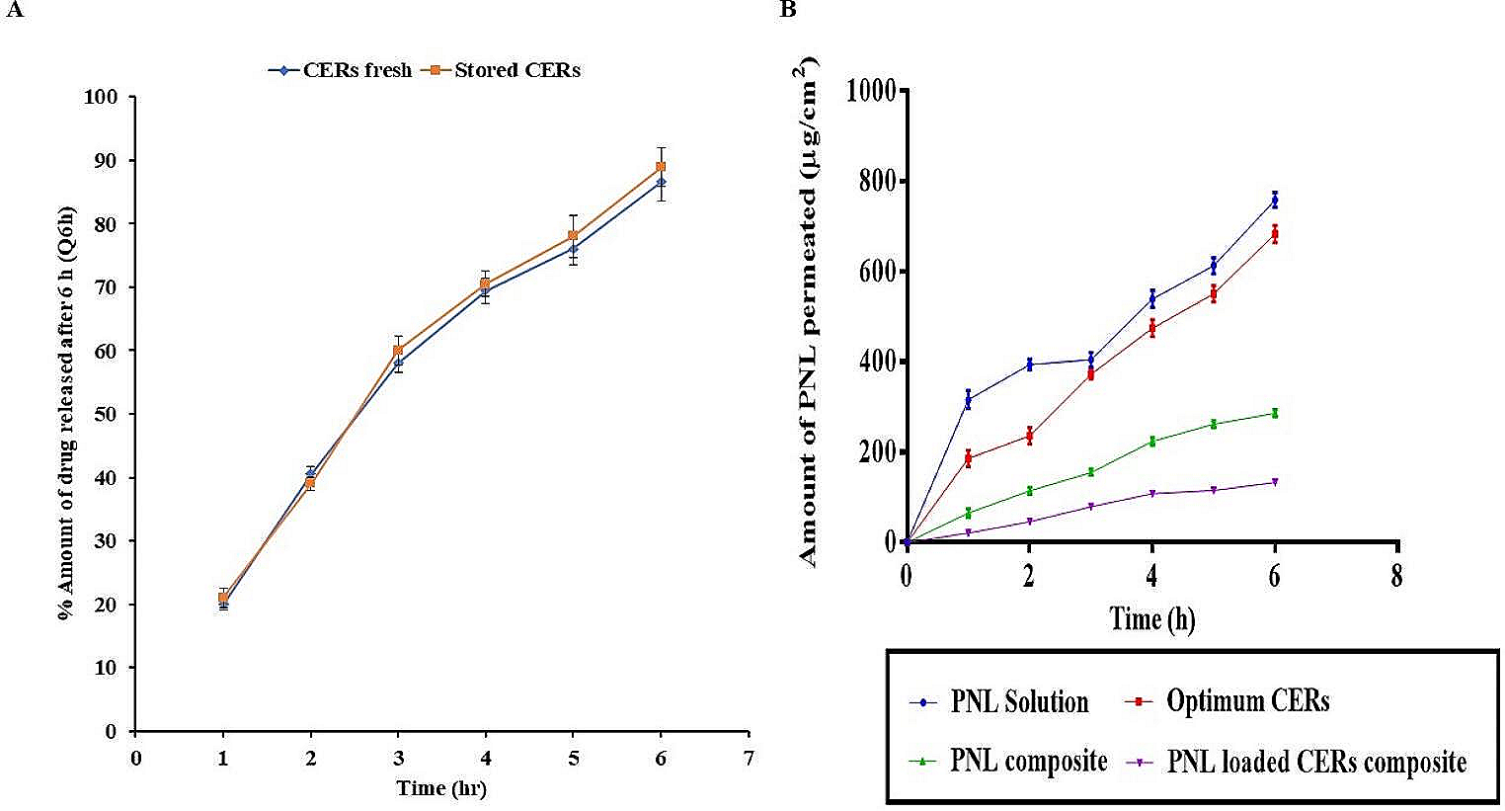
Similarity figure for the fresh and stored optimum CER (**A**), and Ex-vivo permeation profile of PNL from PNL-loaded CERs nanocomposite, PNL-composite, the optimum CER compared to PNL solution (**B**). *Abbreviations* PNL; Propranolol Hydrochloride, and CERs; Cerosomes

At the end of the storage interval (3 months in a refrigerator 4℃), there was no marked variation in the appearance of the optimum CER dispersion. The evaluated physical parameters of the stored CER in comparison to the fresh one with EE% of 91.99 ± 1.01%, PS of 390.00 ± 1.30 nm, PDI of 0.370 ± 0.001, ZP 31.00 ± 0.09 mV, and Q6h (%) of 67.00 ± 0.03% were statistically analyzed and displayed that there was no significant difference (*P* > 0.05) in the EE%, PS, PDI, and Q6h (%). (Fig. 5A) reveals that both fresh and stored CERs showed almost identical release profiles. This observation was proved by the computed value of the similarity factor (*f*_*2*_ = 85.20) indicating that the storage had no remarkable impact on the release of the PNL. The previous findings might be because of the presence of DDAB which potentially helps in the stability of vesicles by increasing the ZP [72].

### Ex-vivo studies

#### Ex-vivo permeation

The application of nano-systems can deliver the drugs to the skin tissues in a very precise manner and enable their sustained release resulting in an extended activity and a possible reduction in adverse effects [73]. It is worth noting that Hirose, et al. suggested for topical *MRSA* infections, the systemic treatment was not often effective. Hence, topical treatment for *MRSA* with controlled release aspects is required [74]. From Fig. 5B, it is obvious that the amount of PNL-permeated from PNL-loaded CERs nanocomposite was significantly (*P* < 0.5) the lowest compared to PNL-composite (PNL-loaded-alginate hydrogel), the optimum CER and PNL solution (Table 5). The significantly lower permeability from PNL-loaded CERs nanocomposite could be explained due to the efficacy of alginate hydrogel to produce a sustainable drug delivery system by increasing the viscosity of the formed system [21]. Regarding the significant (*P* < 0.5) sustained release of PNL-loaded CERs nanocomposite over the whole formulae, this could be attributed to the successful PNL incorporation within the cross-linked-polymer, consequently for drug release to take place, the drug molecules should diffuse firstly through the CERs membrane and then the hydrogel network. Also, the presence of lipids (PC, and ceramide) might successfully encapsulate PNL and provide sustained release, protection, and controlled release profiles [75]. The prior reasons explained the significant sustained release from PNL-loaded CERs nanocomposite and optimum CER compared to PNL- composite and PNL solution. It is worth bearing in mind that the amphoteric aspect of PNL explains its higher permeability profile from PNL solution in comparison to the other formulations. The amount of drug deposited in the skin was in the following order: 13.28 ± 0.94, 26.25 ± 1.88, 42.97 ± 16.63, and 57.04 ± 1.79 µg/cm^2^, for PNL solution, the optimum CER, PNL-composite, and PNL-loaded CERs nanocomposite, respectively. The significant (*P* ≤ 0.5) highest deposition from the PNL-loaded CERs nanocomposite could be related to the hydrogel-loaded CERs that could form a depot from which the drug can be released.

**Table 5:** Permeation Parameters

Formula	Flux, J_ss_ (ug/cm^2^/hr)	Permeation Coefficient, KP (cm/hr)
PNL solution	107.69	0.02153
Optimal CER	107.84	0.02156
PNL-Composite	48.65	0.00973
PNL-Loaded-CERs-Composite	23.22	0.00464

*Abbreviations* PNL; Propranolol Hydrochloride, and CERs; Cerosomes

#### Ex-vivo confocal laser scanning microscopy studies (ClSM)

**Fig. 6:**
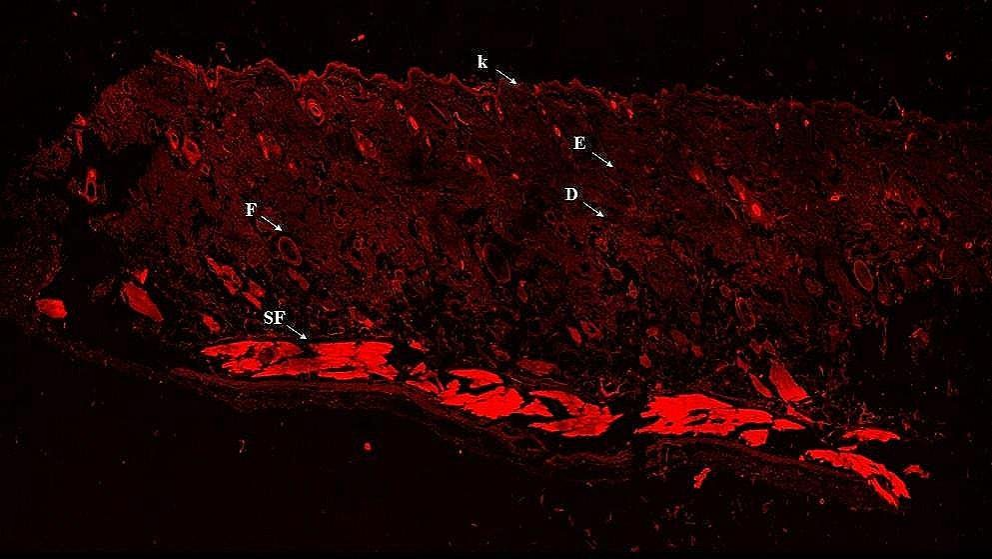
A tile scan confocal laser microscope photomicrograph of a longitudinal section in skin rat tissue treated with FDA-loaded CER. *Abbreviations* FDA; Fluorescein Diacetate, CER; Cerosome, D; Dermis, E; Epidermis, F; Hair Follicles, K; Keratin, and SF; Subcutaneous Fat

The CLSM images showed improved cell internalization and greater drug penetration into the various skin layers which were distinctly displayed in (Fig. 6). The skin treated with the fluoro-labeled optimum CER revealed deep penetration of the dye into the various skin layers. These findings confirmed the accumulation of CERs into deeper skin layers that could be effective for the treatment of *MRSA-related* skin infections. The previous outcomes correlated with Yang et al. during their preparation of CERs for the delivery of methotrexate and nicotinamide for psoriasis management [76].

### In silico studies

#### PNL with affinity towards multiple peptidoglycan-associated MRSA biotargets

The molecular aspects of PNL’s anti-*MRSA* activity were explored by estimating the compound’s binding affinity with several targets involved in *MRSA’s* peptidoglycan biosynthesis. Most of the marketed drugs commonly applied for managing *MRSA* are those designed to hamper its peptidoglycan biosynthesis, the crucial component of the bacterial cell wall [38]. Typically, peptidoglycans confer the bacterial cell wall’s flexibility and robustness and thus interfering with their biosynthesis would mediate bactericidal actions [77]. The presented study explored the potential of PNL to block four multiple steps across peptidoglycan biosynthesis. The first hampered step was mediated by bacterial MurE ligase being involved within the cytosolic biosynthesis of peptidoglycan’s starting units; UDP-*N*-acetylglucosamine for producing the UDP-*N*-acetylmuramyl-multipeptide product [78]. The second target stage was that mediated by the membrane-bound enzyme, MraY, where C55-PyroPhosphate-UDP-*N*-acetylmuramyl-pentapeptide (Lipid I) is formed through catalytic conjugation reaction at the bacterial cytoplasmic side [79]. The third stage involves interpeptide bridging through disaccharide pentapeptide modifications using a pentaglycine chain to be added to the lysine amino acid of the pentapeptide chain. The step is catalyzed by several peptidyl transferases, including FemA. The final stage involved the formation of linear peptidoglycans via the _DD_-transpeptidase catalytic activity of penicillin-binding proteins (e.g. PBP2a) following the transfer of the disaccharide pentapeptides to the cell membrane’s outer surface [80]. The development of multi-target drugs has been considered advantageous for circumventing the most prevalent mechanism of antibiotic resistance which is the target mutations [81, 82]. Out of an evolutionary concept, targeting multiple independent paths for inhibitions is unlikely to allow bacteria to develop resistance over time the thing that would circumvent the pipeline of antimicrobial drug discovery [83].

**Fig. 7:**
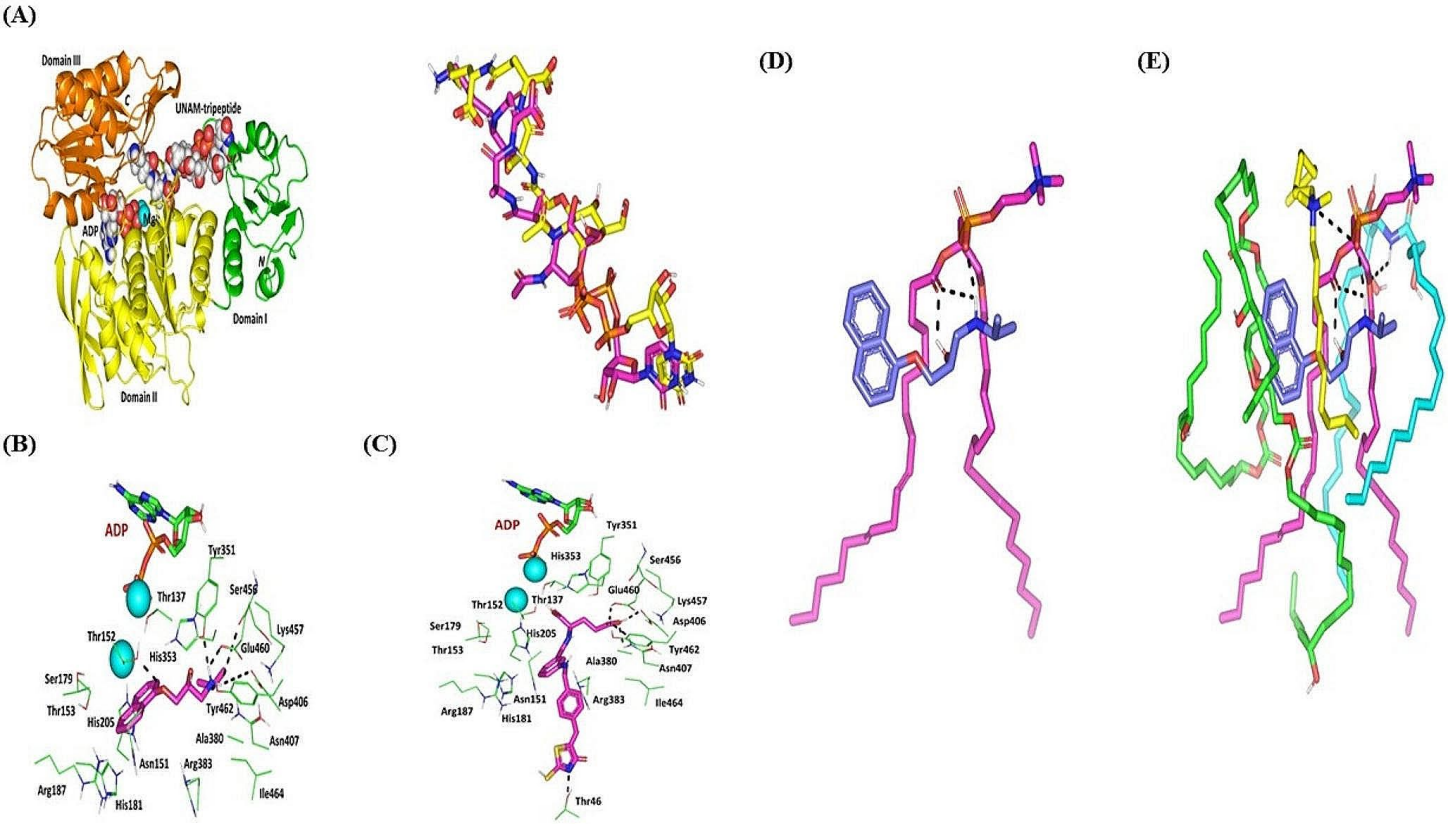
Architecture of *MRSA* MurE and depicted molecular docking poses. (**A**) Upper panel; Cartoon 3D-representation of *MRSA* MurE (PDB; 4c12) ligase showing structural domains; I (Met1–Tyr98; green), II (Pro99–Val332; yellow), and III (Glu333–Asp493; orange) in complex with co-crystallized adenosine diphosphate (ADP), product UDP-N-acetylmuramyl-tripeptide (UNAM-tripeptide), and two magnesium ions (cyan) all as spheres. Letters N and C in bold denote amino- and carboxy terminals, respectively. Lower panel; Aligned redocked MurE product (UNAM-tripeptide; magenta) over its co-crystalline state (yellow). Predicted binding mode of (**B**) propranolol and (**C**) Antibacterial compound T26 as positive reference control. Only surrounding residues within a 5 Å radius as lines are shown and polar interactions are illustrated as black-dash lines. Predicted binding poses of PNL-PC docked complex. Three-dimensional representation of PNL (blue) loaded on (**D**) PC (magenta); (**E**) in combination with nano-formulation additives; ceramide VI (yellow), DDAB (yellow), and kolliphor-RH40 (green). Context-described hydrogen bonding and polar interactions are represented as black dashed lines. *Abbreviations* DDAB; Didodecyldimethylammonium Bromide, PC; Phospholipid, PNL; Propranolol Hydrochloride

The molecular docking of PNL at *MRSA’s* MurE revealed preferential anchoring of the hypertensive compound at the binding domain of the co-crystallized product UDP-*N*-acetylmuramyl-tripeptide. Typically, the product bounds predominantly across the central domain of the ligase protein in proximity to the ATP-binding site (Fig. 7A). Several *MRSA* MurE key residues have been reported as important including Asp406, Ser456, and Glu460, for product/substrate binding and recognition [78], as well as affinity for promising inhibitors [84–86]. Both the negatively charged sidechains of Asp406 and Glu460 as well as the polar mainchain of Ser456 served as the electrostatic trap mediating the stability of UDP-*N*-acetylmuramyl-tripeptide at the binding site. Validation of the docking protocol was highlighted through redocking the co-crystallized ligand under the same adopted parameter, highlighting great superimposed alignment for the co-crystallized and redocked conformation (RMSD = 1.8 Å) (Fig. 7A). Furnishing RMSD below 2.0 Å for co-crystallized ligand relative to its reference conformation/orientation signifies that both the assumed parameters and algorithms were efficient for predicting relevant binding poses, highlighting respective biological significance [87].

Interestingly, PNL showed an extensive polar network with surrounding residues including the ionizable residues, Asp406 (3.1 Å; 121.2°) and Glu460 (2.4 Å; 128.9°), as well as polar Ser456 (2.9 Å; 132.4°) (Fig. 7B). Electrostatic-mediated stability of PNL was further predicted through hydrogen bond interaction between the ligand’s quaternary amine as hydrogen bond donor with nearby Tyr351 mainchain (2.0 Å; 161.8°) as well as ether linker oxygen with Thr152 sidechain (3.0 Å; 134.2°). On the other hand, the PNL aromatic structure was held through hydrophobic and π-mediated contacts (< 5.0 Å) with proximal aromatic residues (His181 and His205) and cationic ones (Arg383). Based on the depicted binding preferentiality, a high docking score was assigned for the anchored compound (–6.3 Kcal/mol). Notably, a thiazolidinylidene-based compound (T26) was adopted as a positive control as a MurE inhibitor with just a higher docking score (–7.2 Kcal/mol) than PNL. The reference compound was reported with high dual inhibition activities towards MurE and MurD from *Staphylococcus aureus* (IC50 = 17.0 µM and 6.4 µM, respectively) and *Escherichia Coli* (IC50 = 180.0 µM and 8.2 µM, respectively) based on radioactivity inhibition assays [88]. Reported studies highlighted the close similarity between MurE secondary structure originating from *MRSA* and *Escherichia Coli* microorganisms (RMSD 1.48 Å along > 450 Cα-atoms and Z-score 21.2) [78, 89]. Furthermore, T26 highlighted great antibacterial activity against *MRSA* and its wild-type strain with MIC of 9.0 µg/ml [88]. Docking of T26 at *MRSA* MurE highlighted dominant electrostatic potentiality guiding its anchoring at the substrate site with interactions with Thr46 (2.6 Å; 159.1°), Asp406 (2.6 Å; 118.4° and 2.8 Å; 115.0°), Asn407 (2.6 Å; 123.3°), and Glu460 (2.0 Å; 140.5°) residues (Fig. 7C). For more information regarding MRSA biotargets please see supplementary materials.

#### Molecular modeling simulation of the nano-formulation drug complex

The magnitude and nature of interaction for PNL with the adopted formulation additives were investigated through a molecular docking study. Throughout the in-silico study, the nature of PC-PNL interaction, in the absence of other formulation additives, was dominated by van der Waal hydrophobic potential. The PNL illustrated favored orientation with its aromatic scaffold towards the PC acyl arms with proximity towards the ester functionalities. On the contrary, the aliphatic arm (PNL amine side chain) depicted favored orientation via the ionizable amino group (*p*Ka = 9.46) towards the negatively charged phosphate head of the PC molecule (Fig. 7D). This favored orientation-mediated triple polar interactions, two via the drug’s ionizable amino group towards the oxygen functionality of the PC (3.34 Å/128.81° and 2.60 Å/152.82°) and a singular one by the drug’s free hydroxyl group (2.29 Å/131.34°). Such depicted compound-PC binding mode and preferential orientation near the phosphate head are consistent with reported studies investigating the binding affinity of small molecules with drug-likeness properties including rosuvastatin, spironolactone, metformin hydrochloride, and levocetirizine dihydrochloride towards the PC molecules [90–92]. Despite the relevant binding interaction for the PNL-PC complex, further stabilization was required since humble docking binding energy was depicted (–3.15 Kcal.mol^− 1^).

Investigating the PNL-PC complex in the presence of the formulation additives depicted more stabilized and favored binding interactions (Fig. 7E). The complex stability was highlighted by utilizing the combined polar and hydrophobic interactions via the formulation additives, ceramide-VI, and DDAB. Both molecules predicted sandwich-like orientation around the PC-PNL complex with their polar heads mainly located near the PC’s phosphate group. The positively charged nitrogen of DDAB and polar NH group at ceramide-VI mediated polar interactions with the PC negatively-charged head (–OPO(O)OH). These predicted hydrogen bonds were considered relevant being at favored bond distances and angles; 2.28 Å/170.90° and 3.56 Å/124.27° for ceramide-VI and DDAB, respectively. On the other side, both DDAB and ceramide-VI depicted extended orientation via their aliphatic hydrophobic arms around the PC’s acyl chains and PNL aromatic scaffold (~ 3.75 Å). This was suggested as beneficial for satisfying the hydrophobic characteristics of the PC-PNL complex. Regarding the docked Kolliphor^®^ RH40 unit, the polymeric molecule depicted favored orientation near the PC acyl arms owing to its linear hydrocarbon chains at repeated intervals. Nevertheless, the free hydroxyl groups at the Kolliphor^®^ RH40 unit showed proximity towards the PNL’s aromatic ring suggesting relevant π-driven non-polar contacts (< 5.00 Å). It is worth noting that Kolliphor^®^ RH40 almost totally shields the PNL’s aromatic scaffold in a way that is highly associated with dominant van der Waal non-polar interactions. Based on all depicted docking orientations, a more stabilized PC-PNL complex was observed following the introduction of the four formulation components mediating an extended network of both electrostatic and hydrophobic potentials. These docking interactions were highlighted with boosted docking energy for PNL (–6.65 Kcal.mol^− 1^) towards the nano-formulation complex. This could be the reason for the improved formulation parameters succeeding the addition of formulation additives serving as carrier agents to mediate the drug loading on the PC molecule for optimized solubilization of the drug.

**Fig. 8:**
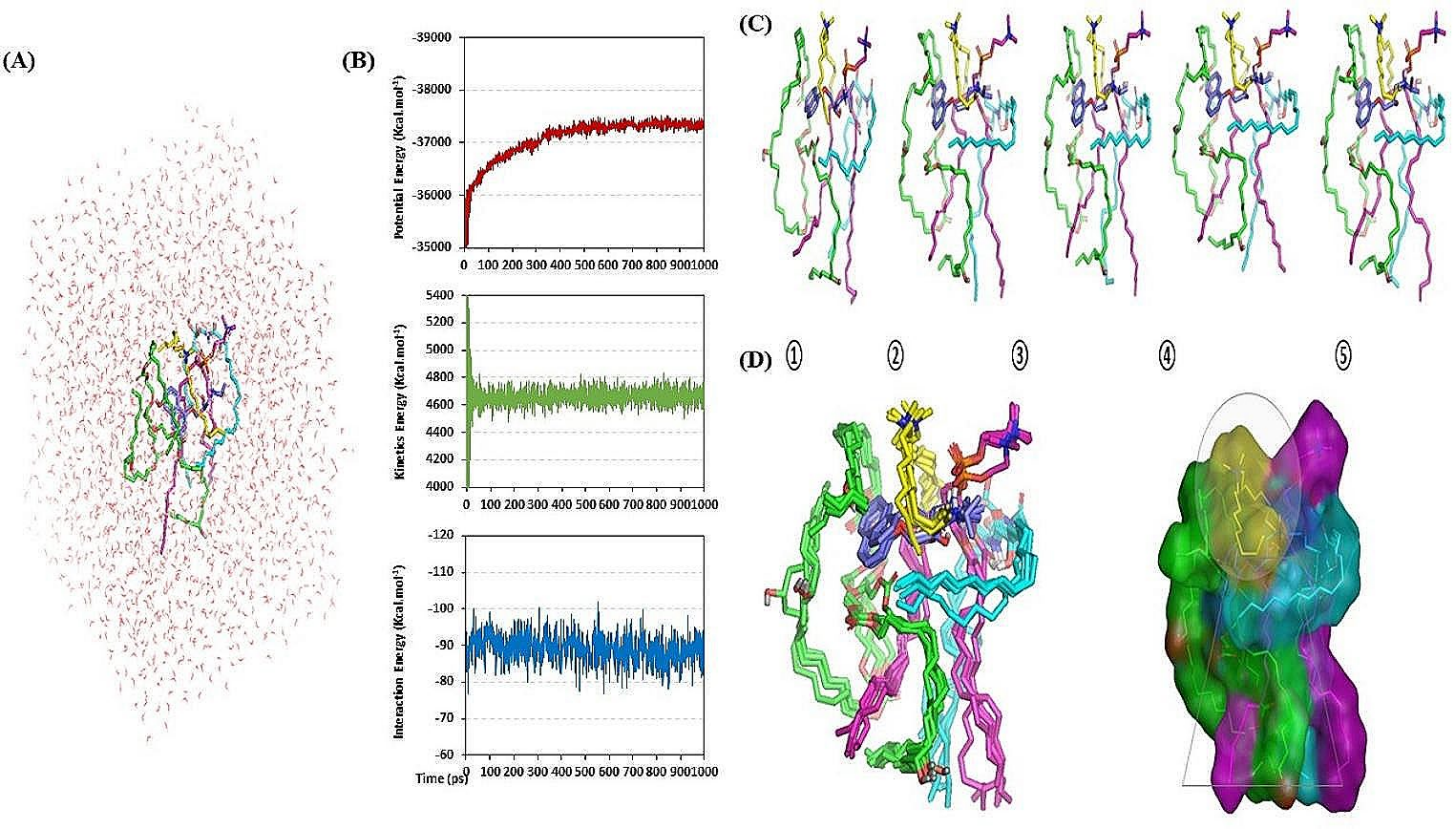
PNL-PC nano-formulation heterocomplex throughout all-atom molecular dynamics simulation within a 100% aqueous solvation system. (**A**) Solvated PNL-PC formulation complex within water cube and ionizable potassium and chloride atoms; (**B**) Plots for the system’s potential and kinetic energies and the drug’s binding-free energy versus the simulated time frames (ps). (**C**) Conformation alterations-time evolution of PNL-PC-formulation additive heterocomplex. Thermodynamic movement formulation components (sticks and differentially colored as previously described) were monitored over simulation trajectories captured at different snapshots ① 200 ps, ② 400 ps, ③ 3600 ps, ④ 800 ps, and ⑤ 1000 ps. (**D**) Overlaid PC-PNL nano-formulation heterocomplex across molecular simulation frames (left panel) and molecular surface 3D-representation of the inverted cone micellar configuration at 100% water solvation system (right panel). Molecular sticks and surface 3D representations were illustrated in colors previously assigned for the optimized formulation components. *Abbreviations* PC; Phospholipid, and PNL; Propranolol Hydrochloride

Thermodynamic stability and dispersion behaviors of the PC-PNL nano-formulation complex in the final solvent of the formulation (100% water) were evaluated through molecular dynamics simulations (Fig. 8A). The whole system stability was highlighted as being relaxed and balanced throughout the monitored kinetic and potential energies across the entire time frame (Fig. 8B). The depicted minimal energy fluctuations with an attained plateau for most of the MD simulation run would confer adequate system stability and convergence. Conformational analysis across extracted time frames (200, 400, 600, 800, and 1000 ps) illustrated significant stability for the simulated PNL-PC nano-formulation complex (Fig. 8C). The PNL maintained its orientation at the PC’s acyl chains till the end of the simulation run (RMSD < 3.00 Å). Moreover, PNL depicted a large negative free-binding energy (average Δ*G* = − 89.26 ± 3.86 Kcal.mol^− 1^) towards the formulation components conferring adequate stability at the nano-formulation complex (Fig. 8D). Ceramide-VI and DDAB formulation additives maintained their enveloped orientation around both the PNL and PC molecules. The polymeric kolliphor^®^ RH40 furnished relevant non-polar binding support for the PC acyl chains as well as the drug’s naphthyl core ring.

Finally, interesting findings were depicted for the PC’s spatial conformation in terms of its lipophilic elongated chains. Conserved polar contacts at the phosphate group made both PC’s hydrophobic acyl tails pull apart. Such thermodynamic behavior depicted an open-compass conformational structure for the PC’s extended tails causing increased volumes and higher solvent-accessible surface areas. On the contrary, smaller solvent-accessible surface areas were maintained throughout the imitation run as the PC complex maintained several robust and dense polar interactions at the phosphate polar head. This depicted packing fashion allowed the PNL-PC nano-formulation complex to acquire an inverted cone structure with maintained micellar configuration being formerly described with various tiny molecules [93, 94] (Fig. 8D).

### In-vitro antibacterial activity

The PNL showed in-vitro antibacterial action against *MRSA USA300* with MIC of 0.625 ± 0 mg/ml. PNL’s antimicrobial efficacy has been assessed against a broad spectrum of microorganisms, The minimum inhibitory concentrations (MICs) of PNL were determined to be 2 mg/mL for *Pseudomonas aeruginosa* and 1 mg/mL for *Serratia marcescens* [95]. The MIC of PNL was evaluated using the agar dilution method, yielding a value exceeding 120 µg/mL, implying its effectiveness against *Staphylococcus aureus, Escherichia coli*, and *Pseudomonas aeruginosa*, as evidenced by growth inhibition zones ranging from 8 to 28 mm [96]. Notably, no previous studies have reported the activity of PNL against *Methicillin-resistant Staphylococcus aureus (MRSA).*

### Anti-biofilm activity of PNL

**Fig. 9:**
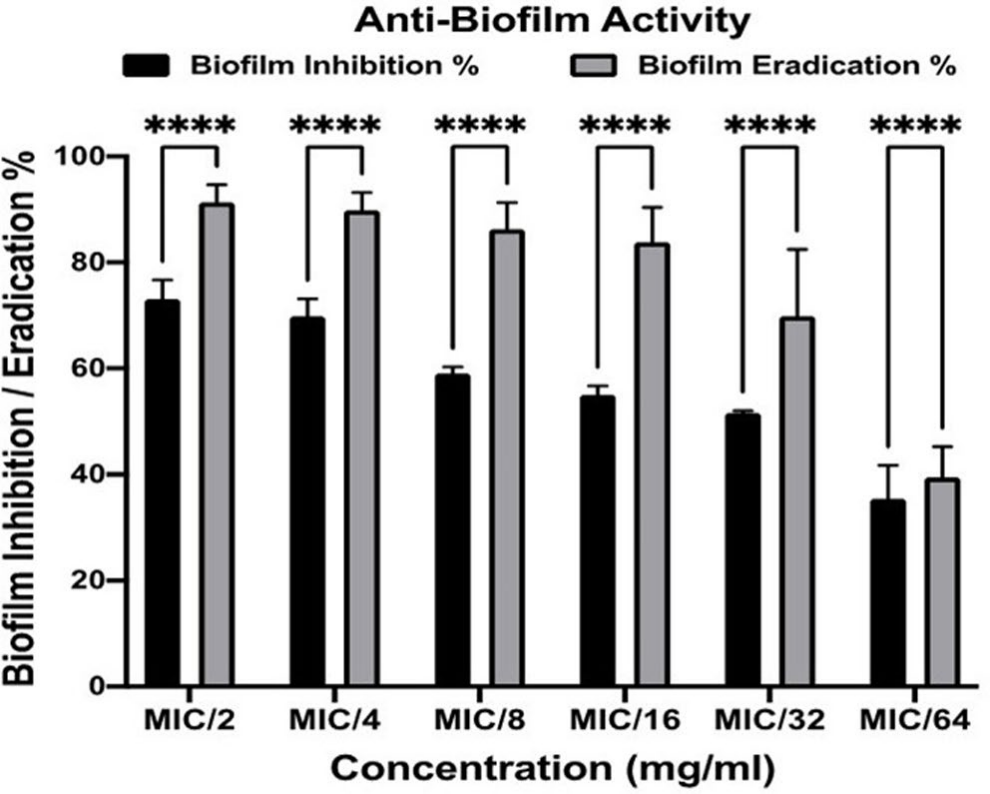
Anti-biofilm activity. Effect of different sub-minimum inhibitory concentrations (MIC) (0.3125–0.0098 mg/ml) of PNL on *MRSA* USA300 biofilm. Results are expressed as mean biofilm inhibition % ± standard error and mean biofilm eradication % ± standard error. **** indicate that the difference is significant at *p* < 0.0001 (two-way ANOVA, Bonferroni’s post-hoc test). *Abbreviations* PNL; Propranolol Hydrochloride

Because of the distinct antibacterial actions of nano-sustained-release materials, they are less likely to cause resistance; thus, they have gained more research attention in treating bacterial biofilm infections. Small-volume and high-surface-area nanomaterials, due to their ability to effectively penetrate biofilms and stick to the deep layers of the infected-tissues, are frequently used as carriers for slow-release antibiotics to produce sustained antibacterial effects [97]. The anti-biofilm activity of PNL was tested at concentrations lower than the established MIC (0.3125–0.0098 mg/ml). PNL significantly hindered the formation of *MRSA USA300* biofilm and significantly eradicated, at all the examined concentrations, the previously formed *MRSA USA300* biofilm (Two-way ANOVA, Bonferroni’s post-hoc test, *P* < 0.05) (Fig. 9). Interestingly, PNL recorded significantly higher biofilm eradication activity at all the examined concentrations (Two-way ANOVA, Bonferroni’s post-hoc test, *P* < 0.0001 (Fig. 9).

### In-vivo studies

#### In-vivo MRSA skin infection model

**Fig. 10:**
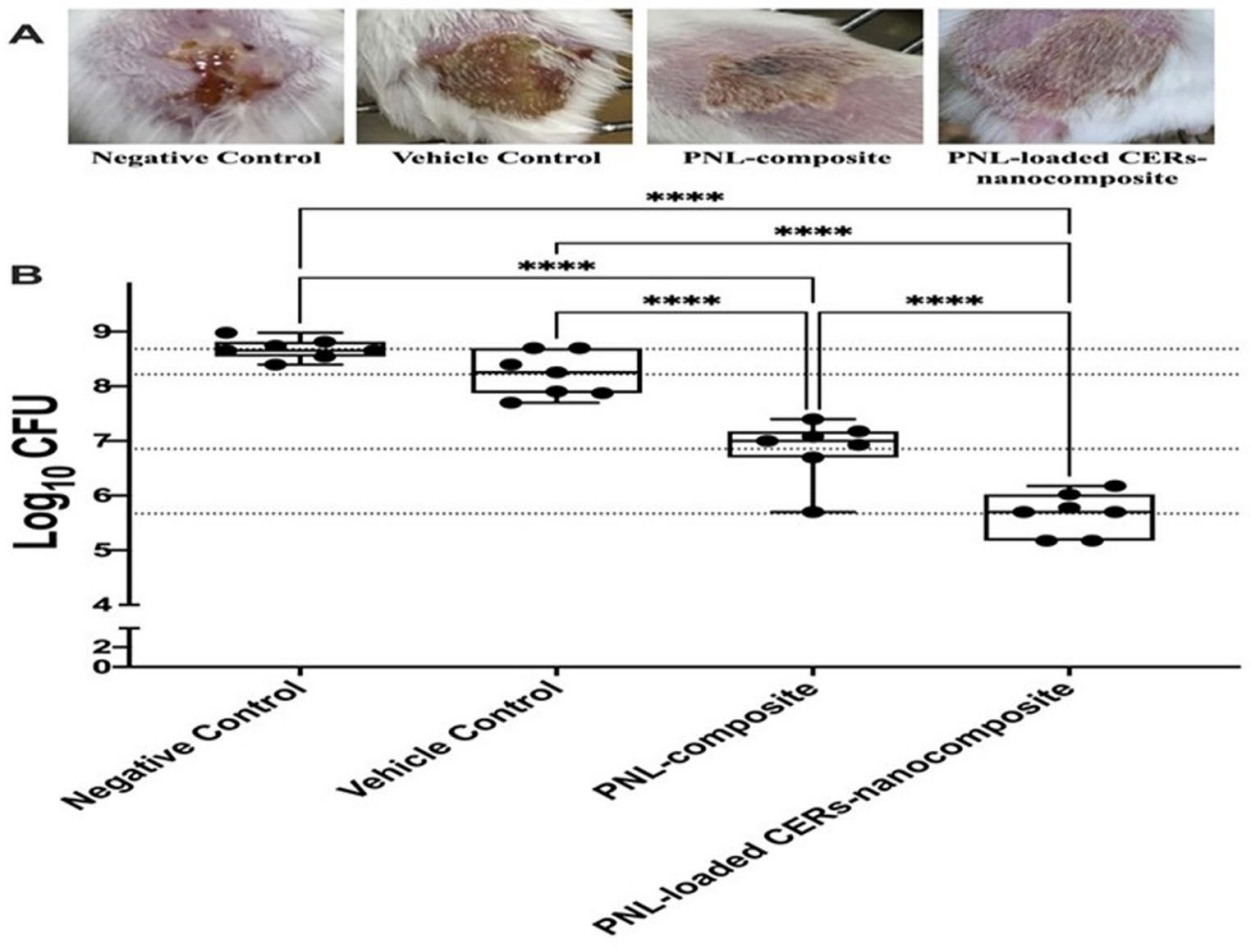
Efficacy of PNL-composite and PNL-loaded CERs nanocomposite in an in-vivo murine model of *MRSA* skin infection. Twenty-eight BALB/C mice were divided into four groups (*n* = 7). A: Photo image of the efficacy of different treatment groups on *MRSA* skin infection in the posterior backs of mice at the end of the experiment. B: Efficacy of different treatment groups on the bacterial load in murine model *MRSA* skin infection. Each data point in the figure represents a mouse. Results are expressed as mean ± standard error. **** indicate that the difference is significant at *p* < 0.0001 (One-way ANOVA, Tukey’s post-hoc test) *Abbreviations* PNL; Propranolol Hydrochloride

The in-vivo antibacterial activity of PNL-composite and PNL-loaded CERs nanocomposite was evaluated against *MRSA* utilizing a murine-infection-model. Four groups of male BALB/C mice (*n* = 7) were intradermally inserted with *MRSA USA300*. After 48 h of infection, the wound appeared at the injection site. PNL-composite and PNL-loaded CERs nanocomposite significantly reduced the count of *MRSA USA300* compared to the negative control group and vehicle control group (One-way ANOVA, Tukey’s post-hoc test, *P* < 0.0001) (Fig. 10). The in-vivo antibacterial activity of the PNL-loaded CERs nanocomposite was significantly greater than that of the PNL-composite (One-way ANOVA, Tukey’s post-hoc test, *P* < 0.0001). The count of bacteria recovered from the PNL-loaded CERs nanocomposite-treated group was 2.543 and 3.008 logs less than that of the vehicle control and negative control groups, respectively. The count of bacteria produced from the PNL-composite-treated group was 1.364 and 1.829 logs less than that of the vehicle control and negative control groups, respectively.

#### Histopathologic evaluation

**Fig. 11:**
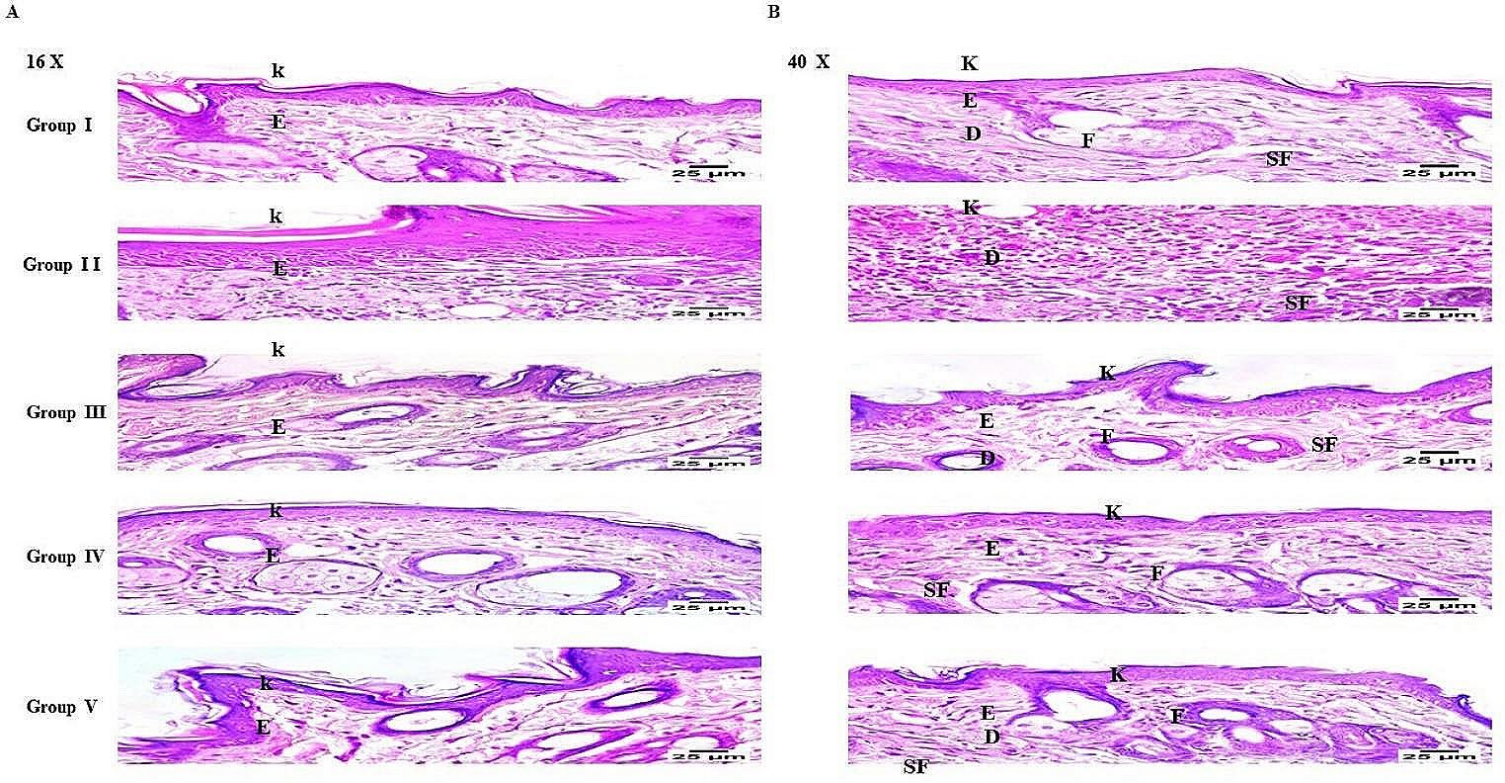
Light microscope photomicrographs showing histopathological sections (hematoxylin and eosin stained) of rat skin normal control (group I), positive control (group II), vehicle-treated group (III), rat skin treated with PNL-composite (group IV), and rat skin treated with PNL-loaded CERs nanocomposite (group V) with a magnification power of 16X to illustrate all skin layers (Left side) and magnification power of 40X (Right side). *Abbreviations* PNL; Propranolol Hydrochloride, CERs; Cerosomes, D; Dermis, E; Epidermis, F; Hair Follicles, K; keratin, and SF; Subcutaneous Fat

The tissue investigation of the five groups (Fig. 11) (group I: negative control, group II: positive control, group III: vehicle control, group IV: PNL-composite, and group V: PNL-loaded CERs nanocomposite). Group I and III photomicrographs showed the normal histological structure of the epidermis and dermis. For group II, the photomicrograph showed an increase in the thickness of the epidermis with infiltration of the dermis by a high number of inflammatory cells. Regarding group IV, the photomicrograph showed infiltration of the dermis by a low number of inflammatory cells. Finally, group V photomicrograph showed the normal histological structure of the epidermis and dermis. Based on the observations made, it appears that the PNL-loaded CERs nanocomposite is safe and unlikely to induce skin irritation in clinical trials that agreed with the in-vivo investigations that did not demonstrate skin intolerability.

## Conclusion

Propranolol hydrochloride (PNL) loaded cerosomes (CERs) were prepared using an ethanol injection technique, and the optimum CER revealed tubular vesicles with excellent EE%, PS, PDI, and ZP. Also, the optimum CER showed good stability for 90 days. Ex-vivo investigations proved the sustained release pattern of PNL-loaded CERs nanocomposite. Furthermore, CLSM images revealed excellent optimum CER deposition into the skin layers. The in-silico investigation confirmed good PNL stability after its incorporation with the other formulation additives. The in-vivo examinations confirmed the efficacy of PNL against *MRSA*. Histopathological examinations revealed that PNL-loaded CERs nanocomposite was safe. These promising outcomes proved the potential application of PNL-loaded CERs nanocomposite as an antibacterial agent against skin infections caused by *MRSA*.

## Electronic supplementary material

Below is the link to the electronic supplementary material.

Supplementary Material 1

## Data Availability

All data generated or analyzed during this study are included in this published article.
