# Supplementary material for: Nanocomposite alginate hydrogel loaded with propranolol hydrochloride kolliphor® based cerosomes as a repurposed platform for Methicillin-Resistant Staphylococcus aureus-(MRSA)-induced skin infection; in-vitro, ex-vivo, in-silico, and in-vivo evaluation
Source: Drug Deliv Transl Res. 2024 May 18;15(2):556–76. doi: 10.1007/s13346-024-01611-z (PMC11683024; doi:10.1007/s13346-024-01611-z)
Supplement: Supplementary file 1 — Supplementary Material 1 [file 13346_2024_1611_MOESM1_ESM.docx]

**Supplementary Materials**

Moving towards the membrane-bound *MRSA* target, PNL depicted relevant anchoring at the protein’s binding site with a docking binding score of –5.7 Kcal/mol within potential energy terms. Typically, the MraY protomer possesses a wide pocket at the cytoplasmic side enclosed in the space between transmembrane (TM) helices 1–5, 8, 9b, and E (Supplementary Figure 1A) [1, 2]. The binding site endorsed the LoopE–TM9b, uridine, uridine-adjacent, lipophilic pockets where polar interactions have been reported as almost dominant for MraY inhibitors at the first three pockets [3, 4]. Interestingly, PNL depicted several strong polar networks at MraY uridine pocket residues; Thr49 (3.1 Å; 120.4°), Asn169 (2.2 Å; 152.9°), and Asp172 (2.4 Å; 127.8° and 2.4 Å; 163.8°) being mediated via the ligand’s polar/ionized functionalities (N^+^H_2_ and free OH) (Figure 12B). The hydrophobic π-π contact with Phe226 (3.7 Å) and van der Waal interaction with His41 (4.1 Å) has further stabilized the PNL binding towards the uridine and LoopE–TM9b pockets, respectively.

Validation of the docking approach was highlighted by adopting a macrocyclic nucleotide antibacterial, namely P5L, as the positive control *S. aureus* MraY inhibitor. The semisynthetic analog of the *Sphaerisporangium sp*-isolated metabolite, sphaerimicin, was reported with strong anti-*S. aureus* MraY inhibition activity (IC50 = 9.1 nM) and MIC against the growth of the gram-positive microorganism (2 μg/mL) [3]. Notably, P5L was crystallized at *Aquifex aeolicus* MraY protein having 1.0 Å alignment RMSD with *MRSA* MraY (Supplementary Figure 1B). Thus, depicting corresponding P5L-pocket orientation/conformation and binding residue patterns at *MRSA* MraY would confirm the docking protocol’s validity. Polar interactions with His41 (2.2 Å; 148.1°), Lys44 (1.6 Å; 120.6°) Asp172 (2.1 Å; 165.4°), Gly173 (3.3 Å), Asp175 (2.4 Å; 137.0°), Asn219 (2.6 Å; 152.7°), and Asp229 (3.1 Å; 127.6°) illustrated the preferential stability of P5L towards the LoopE–TM9b/uridine/uridine-adjacent pockets (Supplementary Figure 1C). At the uridine pocket, strong close-range π-mediated interactions with Phe226 (3.6 Å) were suggested as important for P5L binding stability. Anchoring the P5L’s extended aliphatic chain towards the lipophilic pockets mediates additional stability for the ligand at the MraY binding site. Owing to the larger and more extended architecture of P5L, a higher docking score was assigned with P5L (–8.1 Kcal/mol) the thing that is reasoned for its excellent experimental inhibitory activity with IC50 down to the nanomolar level. Concerning the third stage of peptidoglycan synthesis, PNL affinity towards FemA transferase was assessed to predict the compound’s potentiality to hamper the pentaglycine interpeptide bridging. PNL showed relevant binding affinity (–5.9 Kcal/mol) towards the entrance of the enzyme’s L-shaped catalytic site at domain 1B designated for pentapeptide substrate binding and recognition (Supplementary Figure 2A). The hypotensive drug showed favorable interactions with pocket residues being reported as important for small molecule identification and stability [5]. Polar contacts with Pro151 (2.8 Å; 132.6°), Gln154 (2.9 Å; 125.5°), Tyr328 (2.4 Å; 146.5° and 3.1 Å; 148.7°), and Tyr364 (2.8 Å; 150.5°) were depicted for PNL at optimum hydrogen bond distances and angles (Supplementary Figure 2B). Hydrophobic contacts with non-polar residues such as Phe224, Phe149, Leu153, and Ile155 were also depicted, besides the π-H and π-Cation interactions with respective Gln154 (2.9 Å) and Arg228/Lys33 (~ 3.7 Å) residues. Comparable residue-wise binding patterns were depicted for four FDA-approved drugs being repurposed against FemA through virtual screening [5]. A study by Oselusi, et al. highlighted the polar interactions with Pro151, Gln154, and/or Tyr328 residues being important for the stability of virtual screened hits of natural origins through molecular docking-coupled molecular dynamics simulation analyses. Validation of the docking approach was further proceeded through molecular docking of cyslabdan, the known *S. aureus* FemA inhibitor clinically used for potentiating the imipenem’s anti-*MRSA* activity [6]. The nonantibiotic depicted docking score of (–6.2 Kcal/mol) being mediated through several dominant polar interactions with Leu153 (2.1 Å; 176.7°), Ile155 (2.1 Å; 163.6°), and Tyr328 (3.1 Å; 122.5° and 2.9 Å; 131.1°) besides few hydrophobic contacts (Supplementary Figure 2C).

Exploring the final stage of peptidoglycan synthesis, targeting PBP2a has been considered beneficial for hampering *MRSA* survival. Generally, the catalytic active site located within the PBP2a transpeptidase domain resides at an open groove on the protein surface being available to ligands (Supplementary Figure 3A) [7]. Notably, three conserved motifs were illustrated to cluster around their active sites encompassing the active serine and all the residues required to activate its hydroxyl group for a nucleophilic attack. The first motif comprises the S-X-X-K (Ser403-Thr404-Gln405-Lys406) tetrad where the catalytic serine resides and its sidekick, lysine amino acid can exhibit its vital role for organizing the nearby residues as well as minimizing the p*K*a of the catalytic serine-OH [8]. The second and third conserved motifs consist of the S-X-N (Ser462-Asp463-Asn464) and K-X-G (Lys570-Ser571-Gly572) triads. The characteristic tetrad and triad motifs adopt strikingly similar conformations in a way that makes all active sites within the serine-based PBPs appear just the same [9]. The β-lactam-preventing enzymes (β-lactamases), which are responsible for bacteria resistance through β-lactam catalytic hydrolysis, demonstrate the same three conserved motifs making them mechanistically related to all PBPs. Such outcomes explained how penicillins, cephalosporins, and carbapenems exhibit affinity for several PBPs and β-lactamases, where the latter can confer bacterial resistance. That is why introducing non-β-lactam-based antimicrobial agents, like PNL, to avoid resistance against β-lactam antibiotics is considered highly rationalized [10].

Redocking the co-crystallized cephalosporin antibiotic, ceftaroline [7], provided a validation tool for the adopted docking protocol and algorithm. The novel 5^th^ generation β-lactam drug exhibits broad-spectrum activities, particularly towards the gram-negative bacteria and highly resistant microorganisms including *MRSA*, vancomycin-resistant, intermediate, and heteroresistant vancomycin-intermediate *S. aureus* strains. At the depicted aligned RMSD of 0.5 Å, the redocked ceftaroline managed to replicate its co-crystallized conformation/orientation and residue-wise patterns and achieve a high docking score of –7.6 Kcal/mol (Supplementary Figure 3A). Polar interaction with Ser462 (2.2 Å; 157.4°), Thr600 (2.1 Å; 169.6°), and Glu602 (1.8 Å; 140.3°) were conserved towards the ligand’s polar functionalities of opened β-lactam ring, amidic sidechain, and thiadiazole ring substitution (Supplementary Figure 3B). Stacking between the ligand’s thiazole ring and Tyr446 sidechain through range π-π hydrophobic contact (4.1 Å) provides extra stability near the conserved S-X-N motif. Notably, docked PNL provided comparable residue-wise interaction at PBP2a active site predicting polar interactions with Lys406 (2.2 Å; 152.0°), Ser462 (2.2 Å; 138.5°), and Thr600 (2.5 Å; 122.8° and 2.5 Å; 134.8°) as well as quite displaced π-π contact with Tyr446 at 4.1 Å distance (Supplementary Figure 3C).

**References**

1. Mashalidis EH, Lee SY. Structures of bacterial MraY and human GPT provide insights into rational antibiotic design. J Mol Biol. 2020; 432(18): 4946-4963.

2. Hering J, Dunevall E, Ek M, Brändén G. Structural basis for selective inhibition of antibacterial target MraY, a membrane-bound enzyme involved in peptidoglycan synthesis. Drug Discov Today. 2018; 23(7): 1426-1435.

3. Nakaya T, Yabe M, Mashalidis EH, Sato T, Yamamoto K, Hikiji Y, et al. Synthesis of macrocyclic nucleoside antibacterials and their interactions with MraY. Nat Commun. 2022; 13(1): 7575.

4. Hering J, Dunevall E, Snijder A, Eriksson PO, Jackson MA, Hartman TM, et al. Exploring the active site of the antibacterial target MraY by modified tunicamycins. ACS Chem Biol. 2020; 15(11): 2885-95.

5. Rahman S, Das AK. Integrated multi-omics, virtual screening, and molecular docking analysis of methicillin-resistant staphylococcus aureus usa300 for the identification of potential therapeutic targets: an in-silico approach. Int J Pept Res Ther. 2021; 27(4): 2735-55.

6. Koyama N, Tokura Y, Münch D, Sahl HG, Schneider T, Shibagaki Y, et al. The nonantibiotic small molecule cyslabdan enhances the potency of β-lactams against *MRSA* by inhibiting pentaglycine interpeptide bridge synthesis. PLoS One. 2012; 7(11): e48981.

7. Otero LH, Rojas-Altuve A, Llarrull LI, Carrasco-López C, Kumarasiri M, Lastochkin E, et al. How allosteric control of Staphylococcus aureus penicillin-binding protein 2a enables methicillin resistance and physiological function. Proc Natl Acad Sci USA. 2013; 110 (42): 16808-13.

8. Ghuysen M. Serine β-lactamases and penicillin-binding proteins. Annu Rev Microbiol. 1991; 45(1): 37-67.

9. Frère JM, Page MGP. Penicillin-binding proteins: evergreen drug targets. Curr Opin Pharmacol. 2014; 18: 112-9.

10. Drawz SM, Bonomo RA. Three decades of β-lactamase inhibitors. Clin Microbiol Rev. 2010; 23(1): 160-201.

**Figure Captions**

**Supplementary Figure 1:** Architecture of *MRSA* MarY and depicted molecular docking poses. (A) Upper panel; Cartoon 3D-representation of *MRSA* MarY (AlphaFold; Q2FZ93) membrane-bound catalase showing structural pockets; lipophilic (cyan), uridine (red), uridine-adjacent (green), and LoopE–TM9b (purple). Letters *N* and *C* in bold denote amino- and carboxy terminals, respectively. Lower panel; Overlaid *MRSA* and Aquifex aeolicus MraY proteins. Predicted binding mode of (B) propranolol and (C) antibacterial compound P5L as positive reference control. Only surrounding residues within a 5 Å radius as lines are shown and colored as per constituting pockets. Polar interactions are illustrated as black-dash lines.

**Supplementary Figure 2:** Architecture of *MRSA* FemA and depicted molecular docking poses. (A) Upper panel; Cartoon 3D-representation of *MRSA* FemA (PDB; 1lrz) transferase enzyme showing structural domains; domain 1A (yellow), domain 1B (gray), additional domain 1 (green), and domain 2 (blue). Letters *N* and *C* in bold denote amino- and carboxy terminals, respectively. Lower panel; Predicted binding mode of (B) propranolol and (C) antibacterial nonantibiotic cyslabdan as positive reference control. Only surrounding residues within a 5 Å radius as lines are shown and colored as per constituting domains. Polar interactions are illustrated as black-dash lines.

**Supplementary Figure 3:** Architecture of *MRSA* PBP2a and depicted molecular docking poses. (A) Upper panel; Cartoon 3D-representation of *MRSA* PBP2a (PDB; 3zg0) transpeptidase enzyme in complex with ceftaroline co-crystalline ligand and showing structural domains; transpeptidase domain (blue) and allosteric domain (green-yellow). Letters *N* and *C* in bold denote amino- and carboxy terminals, respectively. Lower panel; Aligned redocked ceftaroline (magenta) over its co-crystalline state (yellow). Predicted binding mode of (B) ceftaroline as positive reference control and (C) propranolol. Only surrounding residues within a 5 Å radius as lines are shown and colored as per constituting domains. Polar interactions are illustrated as black-dash lines.

**Supplementary Figure (1)**


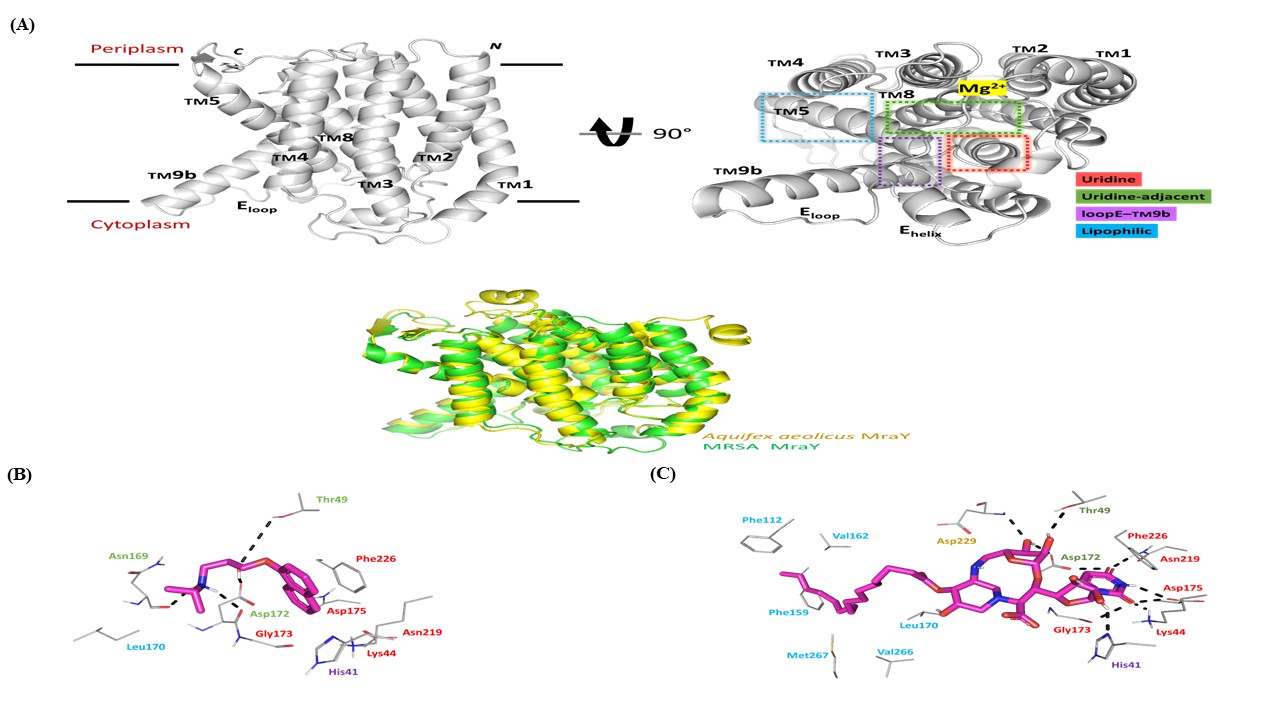


**Supplementary Figure (2)**


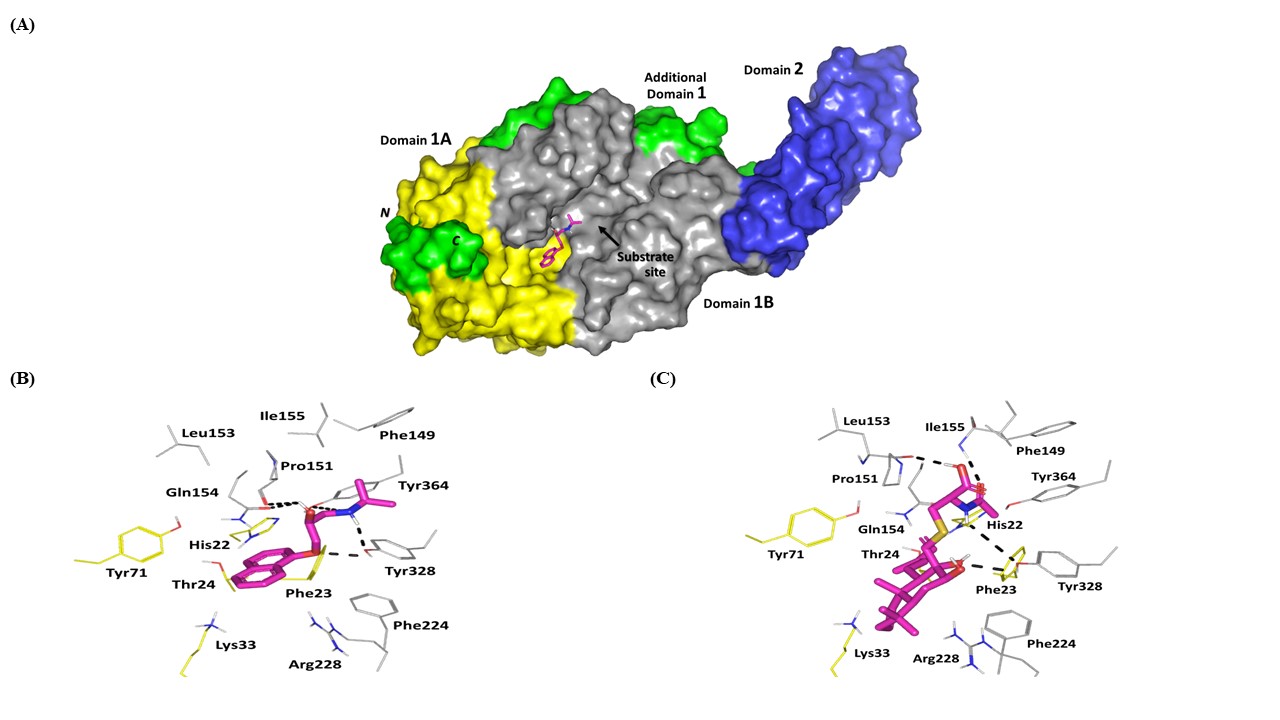


**Supplementary Figure (3)**


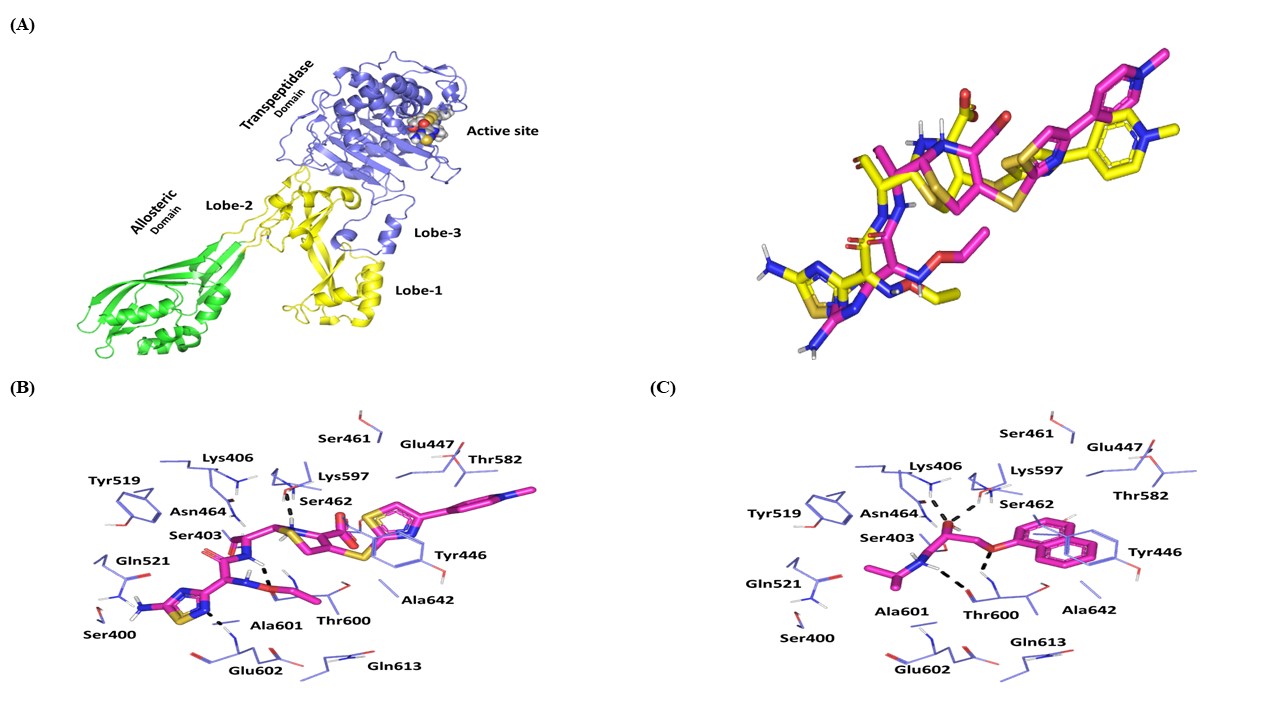


**Supplementary Table (1) Predicted results for CERs.**

| **DDAB amount (mg)** | **Ceramide Type** | **Kolliphor Type** | **EE%** | **ZP (mV)** | **PS (nm)** | **PDI** | **Desirability** |
| --- | --- | --- | --- | --- | --- | --- | --- |
| 0 | IV | Kolliphor RH | 96.566 | 8.663 | 504.968 | 0.363 | 0.793 |
| 0 | III | Kolliphor RH | 92.984 | 4.642 | 467.297 | 0.359 | 0.76 |
| 5 | III | Kolliphor RH | 87.917 | 23.273 | 364.146 | 0.361 | 0.709 |
| 10 | IV | Kolliphor RH | 81.136 | 38.05 | 275.078 | 0.361 | 0.558 |
| 5 | IV | Kolliphor EL | 80.832 | 27.108 | 399.561 | 0.359 | 0.506 |
| 10 | III | Kolliphor RH | 77.179 | 32.66 | 246.087 | 0.362 | 0.448 |
| 0 | III | Kolliphor EL | 84.226 | -4.157 | 566.448 | 0.36 | 0.436 |
| 5 | III | Kolliphor EL | 75.848 | 21.722 | 389.839 | 0.364 | 0.372 |
| 10 | IV | Kolliphor EL | 69.739 | 34.805 | 279.772 | 0.361 | 0.2 |
| 10 | III | Kolliphor EL | 65.151 | 31.29 | 258.233 | 0.366 | 0.009 |
